# One-pot synthesis of trifunctional chitosan-EDTA-β-cyclodextrin polymer for simultaneous removal of metals and organic micropollutants

**DOI:** 10.1038/s41598-017-16222-7

**Published:** 2017-11-17

**Authors:** Feiping Zhao, Eveliina Repo, Dulin Yin, Li Chen, Simo Kalliola, Juntao Tang, Evgenia Iakovleva, Kam Chiu Tam, Mika Sillanpää

**Affiliations:** 1 Laboratory of Green Chemistry, School of Engineering Science, Lappeenranta University of Technology, Sammonkatu 12, FI-50130 Mikkeli, Finland; 2 National & Local Joint Engineering Laboratory for New Petro-chemical Materials and Fine Utilization of Resources, Hunan Normal University, 410081 Changsha, China; 3 Department of Chemical Engineering, Waterloo Institute for Nanotechnology, University of Waterloo, 200 University Avenue West, Waterloo, Ontario N2L 3G1 Canada

## Abstract

The global contamination of water resources with inorganic and organic micropollutants, such as metals and pharmaceuticals, poses a critical threat to the environment and human health. Herein, we report on a bio-derived chitosan-EDTA-β-cyclodextrin (CS-ED-CD) trifunctional adsorbent fabricated via a facile and green one-pot synthesis method using EDTA as a cross-linker, for the adsorption of toxic metals and organic micropollutants from wastewater. In this system, chitosan chain is considered as the backbone, and the immobilized cyclodextrin cavities capture the organic compounds via host-guest inclusion complexation, while EDTA-groups complex metals. The thoroughly characterized CS-ED-CD was employed for batch adsorption experiments. The adsorbent displayed a monolayer adsorption capacity of 0.803, 1.258 mmol g^−1^ for Pb(II) and Cd(II) respectively, while a heterogeneous sorption capacity of 0.177, 0.142, 0.203, 0.149 mmol g^−1^ for bisphenol-S, ciprofloxacin, procaine, and imipramine, respectively. The adsorption mechanism was verified by FT-IR and elemental mapping. Importantly, the adsorbent perform is effective in the simultaneous removal of metals and organic pollutants at environmentally relevant concentrations. All these findings demonstrate the promise of CS-ED-CD for practical applications in the treatment of micropollutants. This work adds a new insight to design and preparation of efficient trifunctional adsorbents from sustainable materials for water purification.

## Introduction

The increasing global contamination of water resources by both inorganic and organic micropollutants, such as heavy metals, pharmaceuticals, and endocrine disrupting chemicals, has become one of the critical environmental problems facing humanity^1–3^. Although most of these pollutants are reported in low concentrations in aqueous environments, they have raised public concerns for the potential negative effects on aquatic ecosystems and human health^4,5^, especially when they are present in complex mixtures^1,6^. Extensive research has been conducted on the removal of a single class of micropollutants individually (either heavy metals or organic micropollutants). However, the heterogeneity of these micropollutants in wastewaters makes removal more challenging due to their diverse chemical properties^7^. Therefore, it is urgent to develop a facile and efficient method to simultaneously remove both the inorganic and organic micropollutants from wastewaters.

Activated carbon has been the most popular adsorbent material for the removal of organic pollutants from wastewater^8^. However, the high cost of this material and usually poor removal of heavy metals^9^ restrict its industrial scale application in the treatment of wastewaters containing both inorganic and organic micropollutants. Moreover, the thermal regeneration of exhausted active carbon is energy consuming and the performance cannot be fully recovered^10^. β-cyclodextrin (β-CD), a natural seven-membered cyclic oligosaccharides produced from starch by enzymatic conversion^11,12^, offer great potential in the adsorptive removal of organic micropollutants from contaminated water^2^. β-CD is well-known for encapsulating appropriately sized organic molecules by the host-guest inclusion complexation^13^, however, its high water solubility hinders its practical application in aqueous systems^14^. Two general approaches have been proposed to address this problem: i) cross-linking of β-CD with cross-linkers such as epichlorohydrin (EPI) which has been reported to have high level of toxicity to human beings and animals^15^, to obtain insoluble CD polymers (CDP)^16,17^ and ii) grafting β-CD via chemical linkers onto insoluble supports^18^. Currently, our group has immobilized β-CD onto the surface of cellulose nanocrystals@Fe_3_O_4_@SiO_2_ for the removal of pharmaceuticals^19^. Chitosan, a linear glucosamine polysaccharide produced from shrimp and other crustacean shells by alkali hydrolysis, has been widely applied as a low-cost adsorbent for heavy metal removal^20^. A series of chitosan grafting with β-cyclodextrin polymers have been developed as adsorbent and drug carrier in a wide range of environmental and medicinal processes^21–23^. Aoki *et al*. prepared an insoluble chitosan polymer bearing β-CD for the adsorption of *p*-nonylphenol and bisphenol A via using 1-ethyl-3-(3-dimethylaminopropyl)-carbodiimide hydrochloride (EDC) as a cross-linker^23^. However, EDC is a ‘zero-length’ cross-linker since the amide linkages are formed without giving a spacer molecule^21^, probably resulting in steric hindrance between β-CD cavities and chitosan moieties^14^. This may limit the inclusion of organic molecules into CD cavities, significantly reducing the adsorption performance of the reported chitosan-graft-CD polymer^15^. Moreover, EDC is also reported to be relatively expensive and not so environmental friendly^24^. Recently, our group has successfully cross-linked chitosan using ethylenediaminetetraacetic acid (EDTA), ethylene glycol tetraacetic acid (EGTA), and diethylenetriaminepentaacetic acid (DTPA) to enhance metal adsorption performance as well as to prevent chitosan from dissolving in acidic environment^25^. Notably, EDTA is a very powerful chelating agent for divalent metals and it has also been reported to be efficiently degraded by catalysts^26^. Thus, EDTA is widely used in industrial processes to prevent metal ion impurities and some undesirable reactions^9^, as well as an important medication in basic health system^27^. The cross-linking method using EDTA has raised concerns since EDTA act as not only a cross-linker but also metal binding sites. More importantly, EDTA is cheaper and less toxic in comparison with conventional cross-linkers, such as EPI and EDC, so it could be an excellent system for the proposed application^24^. More recently, our group has synthesized an EDTA-cross-linked β-CD bifunctional polymer for the simultaneous removal of metals and dyes from wastewater^28^ as well the recovery of Rare Earth Elements (REE) from seawater^29^. This novel material has many important advantages, such as green synthesis and bi-functionalities for specific pollutants, but it also poses challenges for industrial scale application, such as the low yield of the target bifunctional adsorbent from pristine EDTA and β-CD, due to the by-product of water-soluble β-CD oligomers. Moreover, all these reported EDTA-cross-linked polymers were synthesized based on a single component substrate (either chitosan or β-CD), and studies extending the proposed green cross-linking technology for any binary-component substrates have not been reported.

**Figure 1:**
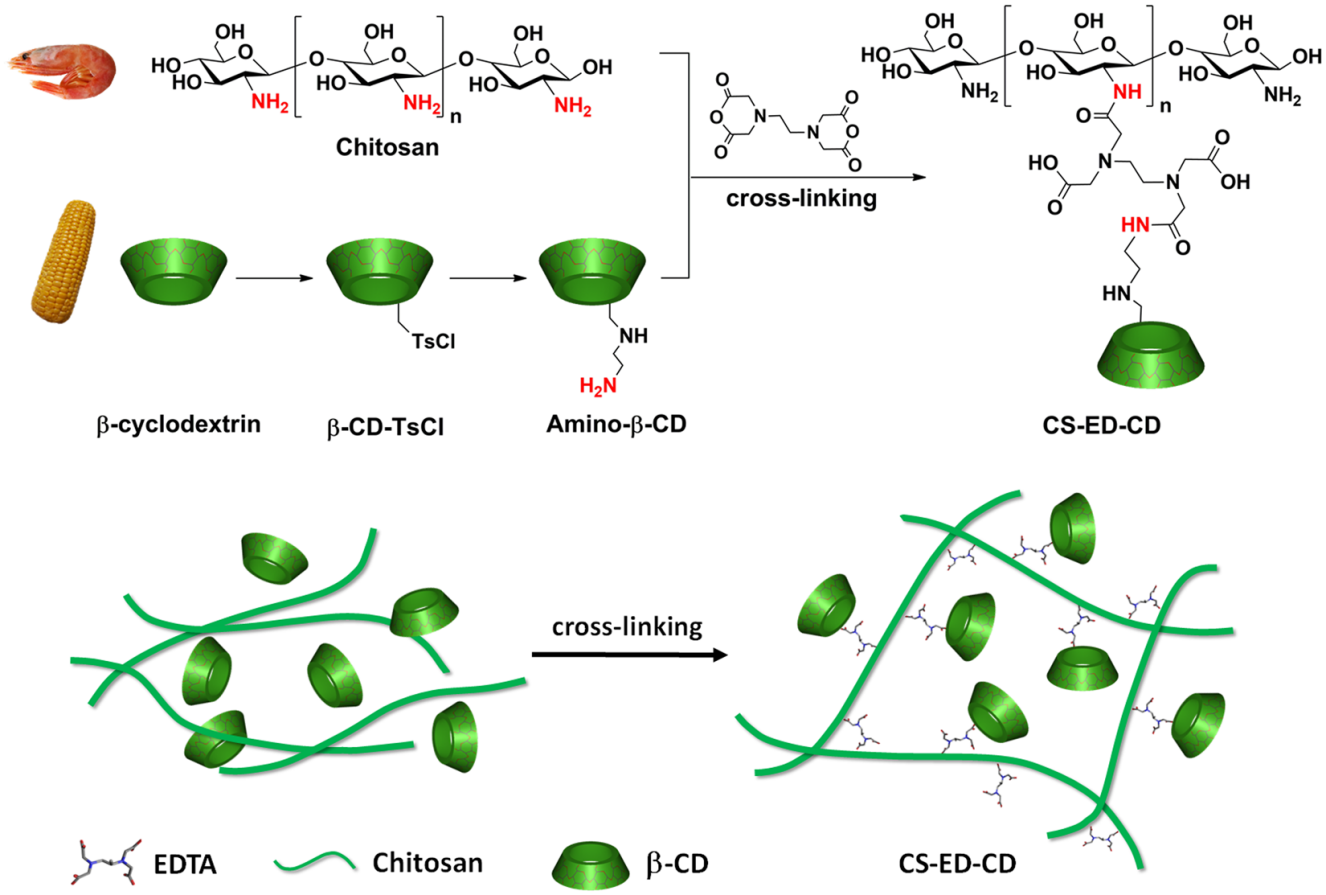
Scheme for one-pot synthesis of CS-ED-CD via EDTA-cross-linking (top), and schematic illustration of the formation of the CS-ED-CD (bottom).

Here we report a one-pot synthesis of an insoluble EDTA-cross-linked chitosan bearing β-CD trifunctional material that can remove both metals and organic micropollutants from aqueous solution. The CD moieties are covalently immobilized onto the chitosan molecules by EDTA linkers (Fig. 1). Each component of the chitosan-EDTA-β-cyclodextrin (CS-ED-CD) has a crucial role in its functioning. The CD cavities are in charge of capturing organic pollutant molecules via the host-guest inclusion interaction. The EDTA moieties are responsible for both cross-linking and heavy metal adsorption. Chitosan is considered as the backbone of this novel polymer and the introduction of chitosan is expected to enhance the loading of β-CD and improve the yield of the water-insoluble adsorbent. Moreover, as a widely used material for heavy metal removal, chitosan is also expected to supplement the adsorption performance for heavy metals. It was reported that lead (Pb) and cadmium (Cd) are the most emergent inorganic micropollutants present in aquatic systems^1^. Thus these two heavy metals and four typical organic micropollutants, bisphenol S (BPS, endocrine disruptor)^2^, ciprofloxacin (CIP, antibiotic)^3^, procaine (anesthetic), and imipramine (antidepressant)^19^, were chosen as model pollutants to evaluate the adsorption ability of CS-ED-CD. Besides the adsorption kinetics and isotherms of each pollutant on CS-ED-CD in single systems, the removal of a complex mixture of inorganic and organic micropollutants at environmentally relevant concentrations (µg L^−1^) and the adsorption mechanism as well as the regeneration of the spent adsorbent were further investigated.

## Results and Discussion

### Characterization and properties of CS-ED-CD

**Figure 2:**
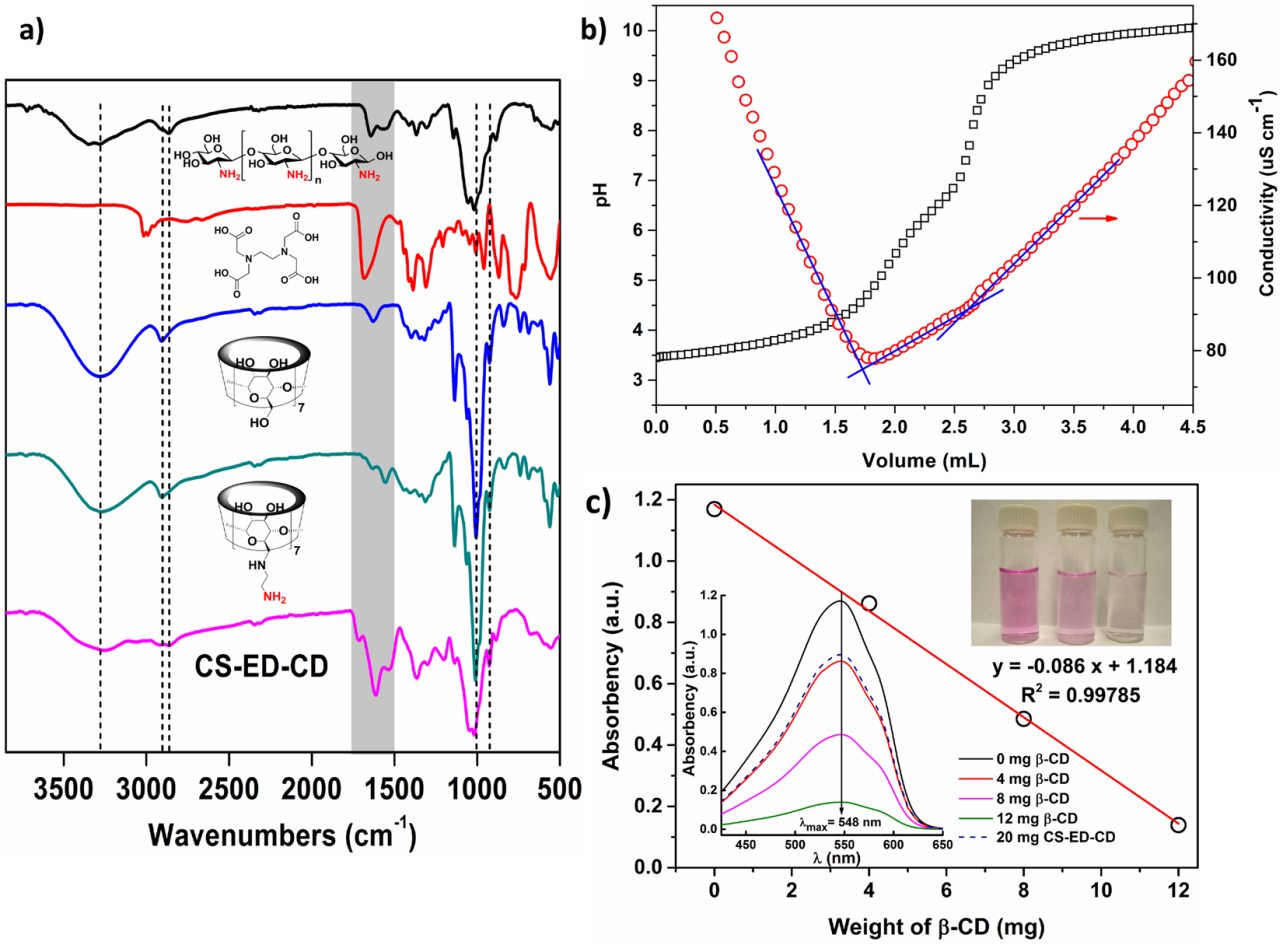
(**a**) FTIR spectra of CS-ED-CD polymer and monomers. Spectra are labelled by chemical structure or compound name (top to bottom: chitosan, EDTA, β-CD, and amino-β-CD); (**b**) simultaneous conductometric-potentiometric titration curves of CS-ED-CD; (**c**) quantitative analysis of active β-CD cavities by photometric titration. Insert, bottom left: UV adsorption spectra of alkaline phenolphthalein solution treated with varying amount of pristine β-CD and 20 mg CS-ED-CD; top right: original alkaline phenolphthalein, treated with 20 mg CS-ED-CD, and treated with 12 mg pristine β-CD (from left to right).

Acid anhydrides such as succinic anhydride react readily with primary amine to yield an amide bond via nucleophilic addition-elimination^30^. Both chitosan and amino-β-CD are substrates containing abundant primary amine groups. Moreover, an EDTA dianhydride can react with two amino groups and act as a cross-linker. In the one-pot synthesis in presence of both chitosan and amino-β-CD, EDTAs form intra- and inter-molecular cross-links with the chitosan chains to produce hydrogels, and they will also conjugate CD moieties onto chitosan backbones^23^ (Fig. 1). There is a probability of forming cross-links between two amino-β-CD molecules (CD-EDTA-CD), hence we will need to perform a control experiment using EDTA dianhydride to react with only amino-β-CD by the same procedure, however, the obtained CD dimer cross-linked by EDTA was found to be water-soluble, which agrees with the literature^31,32^. The yield of the CS-ED-CD and the three controlled materials are presented in Table S1. The yield of CS-ED-CD was much higher than that of EDTA-CD, confirming that the introduction of chitosan in the trifunctional system could successfully enhance the yield as the hypothesis in the introduction section. The yield of EDTA-CD was relatively low, probably because the cross-linking reaction produced numerous water-soluble β-CD oligomers. In the case of CS-ED-CD, the β-CD oligomers could be immobilized on the chitosan backbone, resulting in water-insoluble materials. The introduction of chitosan in the trifunctional material is therefore of great importance in terms of atomic economy. Figure 2a shows the FTIR spectra of the monomers and CS-ED-CD polymer. Notably, the characteristic peak of -OH group on β-CD at 3309 cm^−1^ was shifted to 3261 cm^−1^ for the amino-β-CD, which could be attributed to the new stretching peak of -NH_2_ at about 3200 cm^−1^ that overlapped with the –OH group^33^. Moreover, the new peak at 1552 cm^−1^ could be assigned to the bending vibration of –N–H– group^34^, confirming the successful amination of β-CD. Finally, the spectrum of CS-ED-CD exhibited –OH stretching band at around 3260 cm^−1^, aliphatic C–H stretching near 2914 cm^−1^, C–C/C–O stretching at 1137 cm^−1^, C–O–C stretching at 1016 cm^−1^, and R–1,4–bond skeleton vibration of β-CD at 931 cm^−1^, which are consistent with the characteristic peaks of β-CD^2,35^. From the CS-ED-CD spectrum, C-H bonds in –CH_2_ (*v* = 2914 cm^−1^) and –CH_3_ (*v* = 2875 cm^−1^), asymmetric vibrations of C–O in the oxygen bridge at 1149 cm^−1^, skeletal stretching of C–O–C at 1092 and 1040 cm^−1^ are features of the saccharine structure of chitosan^36^. More significantly, two new vibration peaks at 1616 and 1722 cm^−1^ in the CS-ED-CD spectrum could be ascribed to the carbonyl groups of amides formed and carboxylic groups introduced, respectively^37,38^. Therefore, all these confirmed that CS-ED-CD comprised of a copolymer with tri-functional structures of chitosan, EDTA, and β-CD moieties.

The results for the elemental analysis are summarized in Table S2. The evident difference of nitrogen content between β-CD and amino-β-CD, further confirmed the successful introduction of amine group on β-CD. The molar amount of -NH_2_ group on amino-β-CD was calculated to be 2.843 mmol g^−1^, in the basis of its nitrogen content (3.98 wt.%). The portions of the tri-functional components in CS-ED-CD polymer were estimated based on the difference of N, C, and H contents between CS-ED-CD polymer and its monomers (chitosan, EDTA, and amino-β-CD). As shown in Table S2, CS-ED-CD has the following composition: 20.75% of chitosan, 59.73% of EDTA, and 19.52% of amino-β-CD. The molar amounts of total EDTA and total amino-β-CD groups in CS-ED-CD polymer were further determined to be 2.046 mmol g^−1^ and 0.162 mmol g^−1^, respectively. However, a part of carboxyl functionalities on EDTA were covalently bonded to chitosan or amino-β-CD via amide bonds, thus they may not participate in the adsorption of metal or organic molecules. Moreover, the cross-linking process will also have an impact on the activity of CD cavities. Hence the activities of EDTA and CD cavities on the adsorbent were further quantified by conductometric-potentiometric titration and photometric titration methods^39^ respectively. Conductometric-potentiometric titration has been widely used to determine the amounts of weak acids on polysaccharides^40,41^. As a typical procedure, 8 mg of CS-ED-CD was dispersed in 30 mL of deionized water, followed by adjusting the pH of the solution to ~3 using 0.1 M HCl. Under gentle stirring, the solution was titrated with 0.05 mM NaOH with the simultaneous measurement of conductivity and pH, and the titration curve is shown in Fig. 2b. The three regions from left to right are related to the neutralization of strong acid, neutralization of weak acid, and the addition of excessive base. Based on the titrated amount of NaOH in the second region, the carboxyl amount on CS-ED-CD was determined to be approximately 5.44 mmol g^−1^. By assuming four carboxyl groups on one active EDTA molecule, the molar amount of active EDTA moieties on the adsorbent was estimated to be 1.36 mmol g^−1^, which is lower than the total EDTA content determined from elemental analyses (2.046 mmol g^−1^). This could be attributed to the fact that some of EDTA groups participate as cross-linkers between the polysaccharides^28^. The amounts of active β-CD cavities were quantified by photometric titration using alkaline phenolphthalein as indicator^28,39^: the phenolphthalein molecule could insert into the active β-CD cavities, reducing the color and UV absorbency. The absorbency of phenolphthalein displayed a negative correlation with the weight of β-CD. As shown in Fig. 2c, the weight of active β-CD in 20 mg CS-ED-CD was estimated to be 3.34 mg, based on the absorbency at 548 nm (0.896 a.u.) and the calibration curve. Thus, the amount of active β-CD cavities on CS-ED-CD was calculated to be 0.147 mmol g^−1^, which is in agreement with the total β-CD content (0.162 mmol g^−1^) obtained from elemental analyses. This indicates that the cross-linking did not significantly affect the activity of CD cavities. The amounts of active functional groups obtained were correlated with the adsorption capacities of metals and organic pollutants, and this will be discussed in isotherms section.

**Figure 3:**
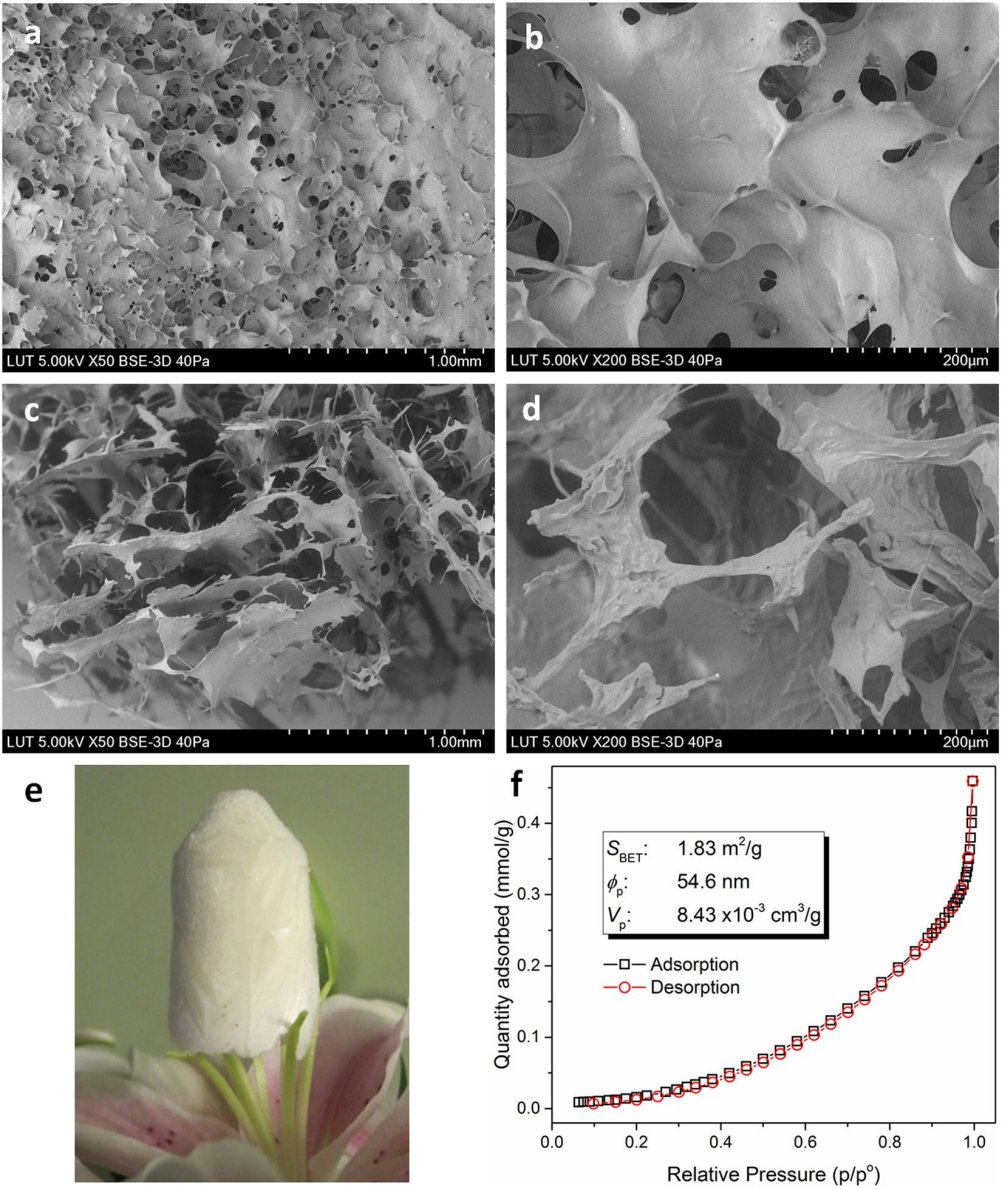
SEM images of freeze-dried CS-ED-CD polymer. Surface morphologies (**a**,**b**) and cross-sectional morphologies (**c**,**d**) of CS-ED-CD hydrogel with varying magnifications; a block of freeze-dried CS-ED-CD hydrogel standing on the stamens of a lilium flower (**e**); and BET isotherm linear plot and BET surface area, average pore diameter and cumulative pore volume data for CS-ED-CD (**f**).

SEM images were acquired to elucidate the microstructure and morphologies of the freeze-dried CS-ED-CD composite polymer (Fig. 3). A thin polymer layer with pores was observed on the surface (Fig. 3a,b), which is related to the collapse of surface pores during the freeze-drying process. The cross-sectional morphologies (Fig. 3c,d) possessed a continuous and porous structure, with pore sizes ranging from 20 to 200 μm. The pores were produced from the ice crystal formation, similar to other natural macromolecular hydrogel structures^42,43^. The freeze-dried hydrogel was very light such that it could be supported on the stamens of a lilium flower (Fig. 3e). The multipoint BET surface area, average pore diameter and DFT cumulative pore volume of CS-ED-CD were examined by BET measurements and the results are presented in Fig. 3f. The CS-ED-CD material has a similar specific surface area and pore volume to the β-CD polymer modified electrospun polyester (PET/CDP) nanofibers reported previously^44^. The porosity measurements are inconsistent with the porous structure observed in SEM, due to the fact that BET measurements determine the pores with diameters less than 250 nm, while the pore sizes in SEM are at µm level. Like most other biopolymer, the specific surface area and porosity of CS-ED-CD were not significantly higher, but this does not affect the removal efficiency toward pollutants, since the removal mechanisms are mainly related to the functional groups on the biopolymers^44^.

**Figure 4:**
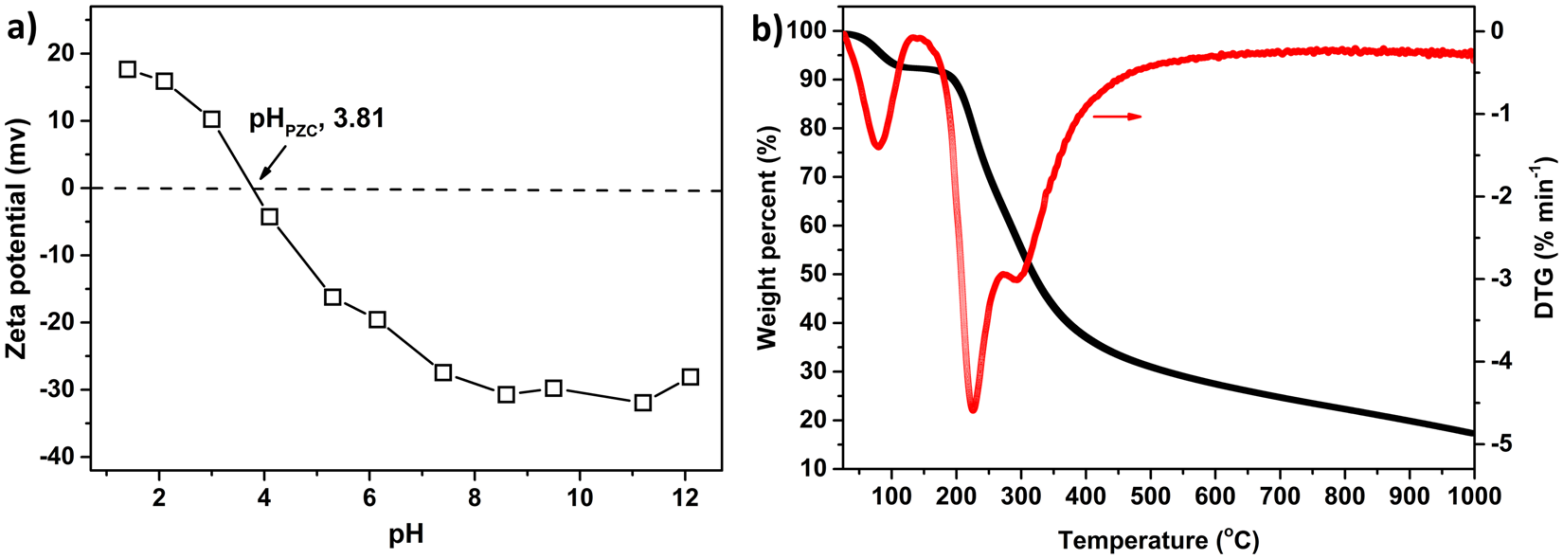
ζ-potential of CS-ED-CD as a function of solution pH (**a**); TGA and DTG curves of CS-ED-CD (**b**).

The ζ-potential of the polymer was measured at different pHs and the results are shown in Fig. 4a. The isoelectric point of CS-ED-CD was approximately 3.8, which is lower than those of pristine chitosan (7.5)^45^ and EPI-cross-linked β-CD (4.42)^28,46^. This could be a result of the introduction of carboxylate groups of EDTA on the surface of CS-ED-CD polymer. The stability of CS-ED-CD polymer was evaluated by thermal analyses (Fig. 4b). Similar to our previous reported EDTA-CD, three thermal transitions at 60–120, 150–270, and 270–1000 °C were observed in the TGA curve of CS-ED-CD, which corresponded to water loss, EDTA decomposition, and the decomposition of polysaccharides (chitosan and CD), respectively^24,28^. The temperatures of water loss and polysaccharide decomposition of CS-ED-CD were slightly higher than those of EDTA-CD, due to the fact that chitosan owns better water-retaining and heat resistance properties than β-CD. Moreover, these three pyrolysis processes were more clearly presented in the DTG curve described by the three peaks at 80.5, 224.5, and 293.5 °C.

### Effect of pH

**Figure 5:**
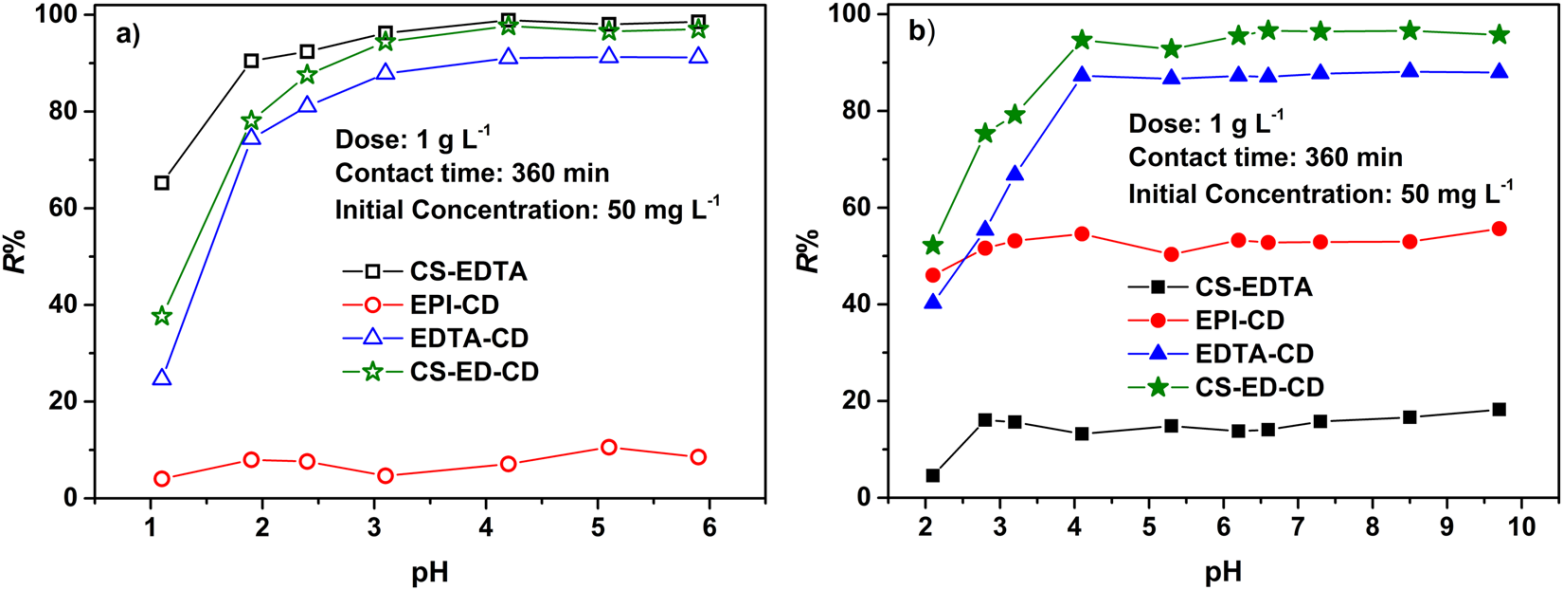
Effect of pH on adsorption of Pb(II) (**a**) and BPS (**b**) by as-prepared CS-ED-CD and controlled adsorbents.

It is well known that the adsorption of pollutants from water are dependent on the solution pH, which controls the surface charge of the adsorbents as well as the ionization degree of the pollutants^24^. Alkaline solutions were not tested for metal to avoid the formation of metal hydroxides (Visual MINTEQ ver. 3.0). Figure 5 and Fig. S1 show the effect of pH on the removal of metals and organic pollutants by CS-ED-CD and other controlled adsorbents. Resembling that of CS-EDTA^45^, the ζ-potential of CS-ED-CD decreased with increasing pH and it possessed a relatively low isoelectric point of 3.8 (Fig. 4a). Thus at pH < 3.8, the surface of CS-ED-CD was positively charged, repelling the metal ions and cationic organic molecules. With increasing pH, the decrease in the surface potential reduced the electrostatic repulsion and enhanced the electrostatic attraction, resulting in the rise of the removal efficiency. At pH above 3.8, the surface of CS-ED-CD became negatively charged and favored the adsorption. It is important to note that CS-ED-CD showed excellent adsorption efficiency of heavy metals, in stark contrast to EPI-CD, confirming that EDTA functional groups not only act as cross-linkers but also as adsorption sites for metal ions. The adsorption efficiency of heavy metals by CS-ED-CD was slightly lower than that of CS-EDTA, and this may be attributed to the higher EDTA amount on CS-EDTA compared to CS-ED-CD^38^. However, CS-ED-CD displayed much better adsorption performance than CS-EDTA for organic pollutants. This could be ascribed to the successful grafting of CD moieties on the polymer chains. Moreover, CS-ED-CD has substantially higher adsorption efficiency for organic pollutants than EPI-CD at pH range of 3–8 due to two reasons: (1) the electrostatic interactions between EDTA-groups and cationic BPS promoted the adsorption, and (2) probably more importantly, the EDTA-functionalization has endowed the nonpolar CD cavities more polar property and that favors inclusion interaction with cationic organic molecules^47^. Notably, the as-prepared CS-ED-CD also showed better removal efficiency than EDTA-CD for both metals and organic pollutants, attributed to the introduction of chitosan, which has been widely used in the adsorption of metals, dyes, and pharmaceutical pollutants^20,24^. The effect of pH on the adsorption of CIP by CS-ED-CD was different from other organic pollutants due to its zwitterionic behavior (Fig. S1b). The CIP removal efficiency increased with pH and reached a plateau at pH 4 to 6, and then decreased with increasing pH. This is because pH affects not only the surface charge of the adsorbent but also the speciation of CIP, which has two pK_a_ values (pK_a1_ 6.1; pK_a2_ 8.7), possessing positive charges at pH < 6.1 and negative charges at pH > 8.7^48^. Thus when the solution pH exceeded 6.1, the CIP was zwitterionic or anionic, thereby reducing the interaction with negatively charged CS-ED-CD adsorbent^49^.

### Adsorption kinetics

**Figure 6:**
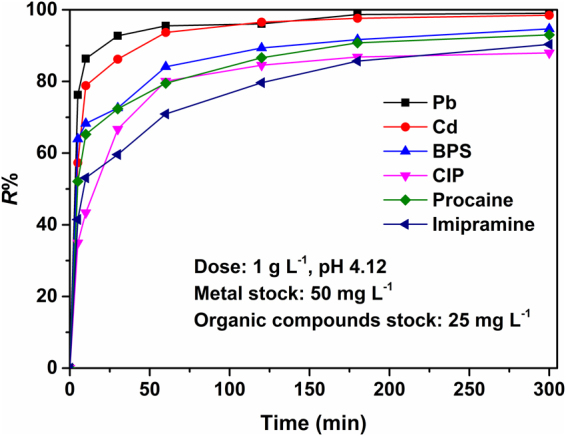
Effects of contact time on uptake of each pollutant by CS-ED-CD.

The effect of contact time on the uptake of each pollutant by CS-ED-CD is presented in Fig. 6 and the UV-vis spectra of four organic pollutants at varying contact time are shown in Fig. S2. In general, the adsorption rates were found to be rapid, attaining 35–75% of adsorption equilibrium uptake within the first 5 min, approaching an equilibrium after 60 min for metals and 180 min for organic pollutants, respectively. Thus, an excessive contact time of 360 min was chosen for the subsequent adsorption experiments. Moreover, for the purpose of investigating the kinetic mechanism of the adsorption process, pseudo-second-order model was applied as follows^50^.

1 $$\frac{{\boldsymbol{t}}}{{{\boldsymbol{q}}}_{{\boldsymbol{t}}}}{\boldsymbol{=}}\frac{{\bf{1}}}{{\boldsymbol{(}}{\boldsymbol{k}}{{\boldsymbol{q}}}_{{\boldsymbol{e}}}^{{\bf{2}}}{\boldsymbol{)}}}{\boldsymbol{+}}\frac{{\boldsymbol{t}}}{{{\boldsymbol{q}}}_{{\boldsymbol{e}}}}$$

where *q*_*t*_ and *q*_e_ (mg g^−1^) are the sorption capacities at time *t* and at equilibrium, respectively, whereas *k* (g mg^−1^ min^−1^) is the rate constant. As shown in Fig. S2, the pseudo-second-order model gave the perfect fit to the kinetic experimental data of CS-ED-CD toward both metals and organic pollutants. Accordingly, the kinetic parameters and correlation coefficient *R* ^2^ values were determined by linear regressions and presented in Table 1. Both the *R* ^2^ values greater than 0.996 and the excellent agreement between the calculated *q*_e_ (*q*_*e,cal*_) and experimental *q*_e_ (*q*_*e,exp*_), clearly indicated that the pseudo-second-order model can describe the sorption kinetics of the process. Interestingly, the metals displayed faster kinetics (higher *k* values, Table 1) than the organic compounds, probably suggesting that there are much more abundant adsorption sites for metal ions than those for organic pollutants on CS-ED-CD^9^. Pb(II) had a faster kinetic compared to Cd(II), which may be due to the smaller hydration number of Pb(II)^25^. The kinetic constants of organic pollutants followed the order of BPS > procaine > CIP > Imipramine, almost consistent with the order of their molecule sizes (Fig. S2, Table 1).

**Table 1:** Parameters of adsorption kinetics of pollutants onto CS-ED-CD fitted by pseudo-second-order model.

Pollutants	*q*_*e,exp*_ (mg g^−1^)	*q*_*e,cal*_ (mg g^−1^)	k (10^−3^) (g mg^−1^ min^−1^)	*R* ^2^
Pb(II)	49.513	49.801	9.067	0.99989
Cd(II)	49.241	49.826	5.408	0.99996
BPS	23.668	23.987	6.359	0.99913
CIP	21.990	22.732	4.692	0.99983
Procaine	23.250	23.674	5.503	0.99903
Imipramine	22.586	23.202	3.281	0.99669

### Adsorption isotherms

To learn more about sorption characteristics of metals and organic pollutants on CS-ED-CD, two typical isotherms, i.e., Langmuir and Sips (Langmuir-Freundlich) models, were used to fit the experimental equilibrium data. The classical Langmuir isotherm, which is based on the assumption of a monolayer adsorption on a homogeneous adsorbent surface with finite and equal affinity sorption sites toward adsorbate^50^, is expressed as follows.

2 $$\,{{\boldsymbol{q}}}_{{\boldsymbol{e}}}=\frac{{{\boldsymbol{q}}}_{{\boldsymbol{m}}}{{\boldsymbol{K}}}_{{\boldsymbol{L}}}{{\boldsymbol{C}}}_{{\boldsymbol{e}}}}{1+{{\boldsymbol{K}}}_{{\boldsymbol{L}}}{{\boldsymbol{C}}}_{{\boldsymbol{e}}}}$$

**Figure 7:**
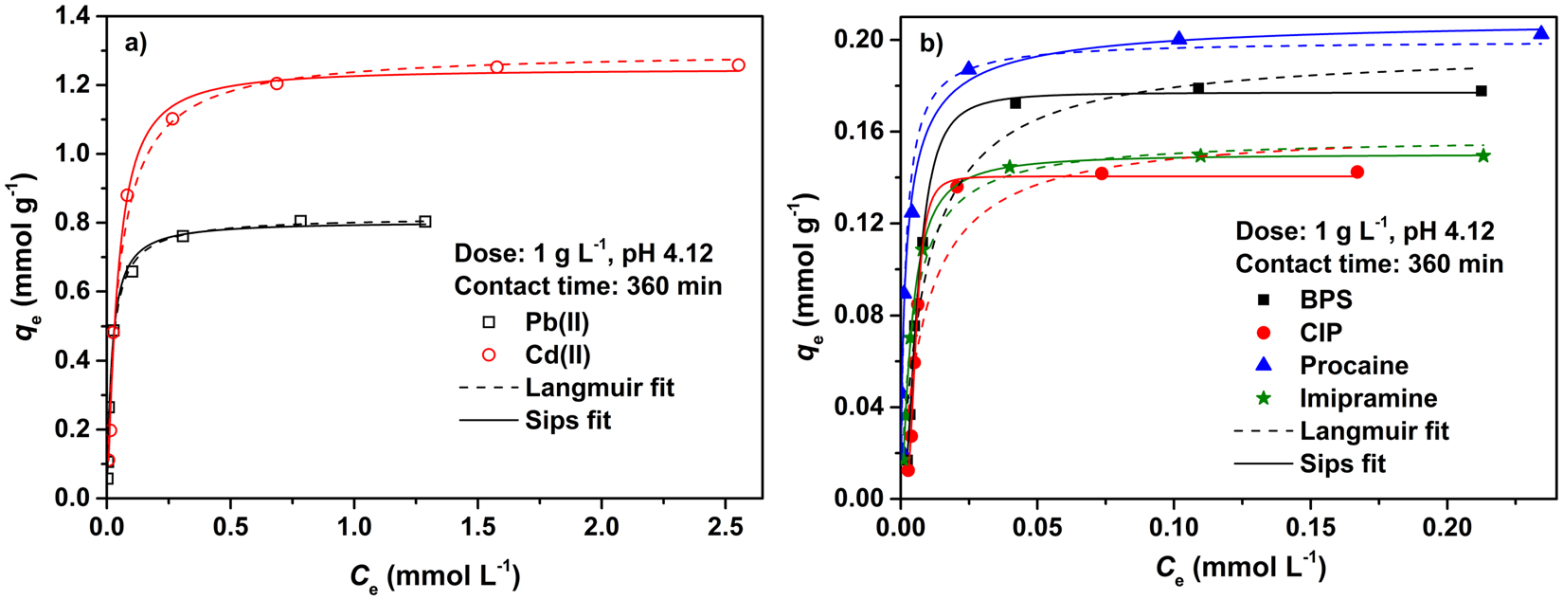
Adsorption isotherms of metals (**a**) and organic pollutants (**b**) onto CS-ED-CD, fitting by Langmuir and Sips models.

The Sips model, which is a combination of the Langmuir and Freundlich models and takes heterogeneity into consideration^28^, is expressed as follows.

3 $${{\boldsymbol{q}}}_{{\boldsymbol{e}}}=\frac{{{\boldsymbol{q}}}_{{\boldsymbol{m}}}{({{\boldsymbol{K}}}_{{\boldsymbol{S}}}{{\boldsymbol{C}}}_{{\boldsymbol{e}}})}^{1/{{\boldsymbol{n}}}_{{\boldsymbol{S}}}}}{1+{({{\boldsymbol{K}}}_{{\boldsymbol{S}}}{{\boldsymbol{C}}}_{{\boldsymbol{e}}})}^{1/{{\boldsymbol{n}}}_{{\boldsymbol{S}}}}}$$

where *q*_e_ (mmol g^−1^) and *C*_e_ (mmol L^−1^) are the adsorption capacity and equilibrium concentration of the adsorbate respectively, while *q*_m_ (mmol g^−1^), *K*_L_/*K*_S_ (L mmol^−1^), and *n*_s_ are the maximum sorption capacity, the energy constant, and the heterogeneity factor attained from nonlinear fitting of experimental data, respectively. The Langmuir and Sips fits are shown in Fig. 7a,b, and the regression parameters for the isotherm models for all pollutants are summarized in Table 2.

**Table 2:** Isotherm parameters of Langmuir and Sips models for metals and dyes adsorption.

Pollutants	*q*_*m,exp*_ (mmol g^−1^)	Langmuir model			Sips model			
		*q*_*m*_ (mmol g^−1^)	*k*_*L*_ (L mmol^−1^)	*R* ^2^	*q*_*m*_ (mmol g^−1^)	*K*_*S*_ (L mmol^−1^)	*n*_*S*_	*R* ^2^
Pb(II)	0.803	0.817	47.252	0.9893	0.793	50.832	1.099	0.9885
Cd(II)	1.258	1.269	20.219	0.9956	1.247	23.487	1.285	0.9758
BPS	0.177	0.196	108.847	0.9335	0.177	162.623	2.241	0.9965
CIP	0.142	0.161	112.195	0.8629	0.141	180.750	3.501	0.9968
Procaine	0.203	0.199	600.716	0.9809	0.210	484.098	0.750	0.9936
Imipramine	0.149	0.158	214.528	0.9821	0.150	252.313	1.409	0.9994

It is observed that metals (Pb and Cd) uptake onto CS-ED-CD could be better described by Langmuir model, displaying a higher correlation coefficient *R* ^2^ values and better curve fitting to experimental data (Fig. 7a) compared to the Sips model. Besides, the calculated *q*_m_ values determined from the Langmuir model were closer to the experimental values (Table 2), suggesting a homogeneous distribution of active adsorption sites (EDTA-groups) for metals on CS-ED-CD^51^. In the case of organic pollutants uptake, conversely, the Sips model gave a much better fit to the experimental data than the Langmuir model (Fig. 7b). Moreover, both the higher *R* ^2^ values and the between agreement between the experimental and calculated *q*_m_ values obtained by Sips model, indicated heterogeneous active sites (CD cavities and EDTA-groups) for organic pollutants on the adsorbent^28^. In addition, the resulting heterogeneity exponent *n*_S_ values of organic pollutants were not equal to unity confirming the heterogeneous adsorption^38^. The higher adsorption affinity *K*_L_/*K*_S_ values of organic pollutants compared to metals revealed that CS-ED-CD appeared to display higher affinity toward organic pollutants^16^.

Remarkably, the maximum sorption capacities (Table 2) corresponded to the capture of 0.590 Pb(II) ion and 0.925 Cd(II) ion per active EDTA group in the adsorbent, while for organic compounds corresponded to the capture of 1.200 BPS, 0.966 CIP, 1.381 procaine, and 1.014 imipramine molecule per active CD cavity, indicating the accessibility of the functional groups in CS-ED-CD^51^. The capture of organic compound molecules per CD group ≥ 1 presumably suggested that chitosan and EDTA groups were also involved in the uptake of organic compounds^2,52^. This behaviour could also be seen in Fig. 5b that about 15% BPS could be removed by CS-EDTA. The results were consistent with the heterogeneous adsorption of organic compound predicted by Sips model.

### Adsorption mechanisms

**Figure 8:**
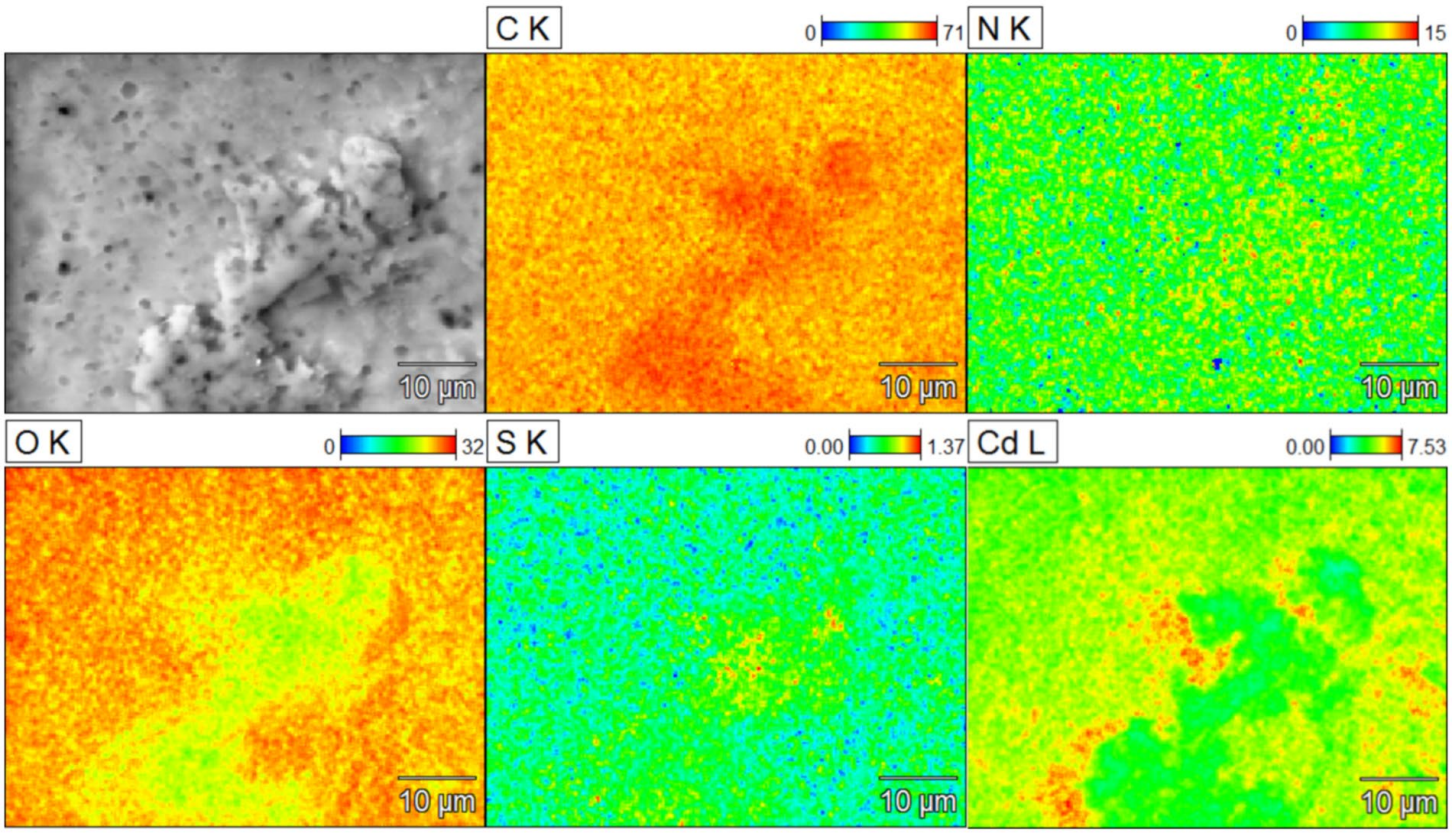
SEM image and the EDS elemental distribution mapping of CS-ED-CD after simultaneous adsorption of Cd(II) and BPS (initial concentration: 100 mg L^−1^). The element of S acts as the indicator of BPS since only BPS contains sulphur. The signal spots of sulphur correlated with those of carbon and nitrogen. The distribution of Cd(II) coincided with the signal spots of oxygen, especially in the area of low oxygen signal there was lower Cd(II) loading.

The elemental distribution for CS-ED-CD after simultaneous adsorption of Cd(II) and BPS is presented in Fig. 8. The colored elemental signal spots depict that the sulfur and cadmium were distributed over the whole surface of CS-ED-CD, indicating the successful load of Cd(II) and BPS (sulphur is from BPS) and the well-distributed adsorption sites on CS-ED-CD. Moreover, the elemental mapping clearly shows the higher amount of Cd than that of S on the adsorbent, which is in good agreement with the maximum adsorption capacity results obtained from the isotherms. Importantly, it is noticed that the distribution of sulphur (BPS) coincided with the signal spots of carbon and nitrogen, indicating the affinity between BPS and amino-β-CD (β-CD-(NHCH_2_CH_2_NH_2_)_1.72_) groups. The correspondence between Cd(II) and oxygen revealed the roles of –COOH in the metal coordination. To gain more insights into the adsorption mechanism, the FT-IR spectra of CS-ED-CD before and after the adsorption of Cd(II) and/or BPS, were compared in Fig. S3. In the case of BPS and BPS-Cd(II) adsorption systems, it is clearly evident that the –OH bond at 3260 cm^−1^ appeared as a wide, broad peak in the range from 3407 to 3241 cm^−1^, which could be ascribed to the introduction of phenolic hydroxyl group of BPS. Similar behavior has also been reported for BPA adsorption^53^. Moreover, the characteristic peaks of BPS, such as aromatic ring stretch at 1583 cm^−1^ and two peaks at 1134 and 1099 cm^−1^ corresponding to sulfonyl groups, were observed in the spectra of the BPS and BPS-Cd(II) adsorptive adducts, indicating that BPS formed host-guest complex with the CD^54^. Similar FT-IR results for the CD/organic molecules were previously reported^28,55^. After Cd(II) adsorption, the peak at 1616 cm^−1^ was bathochromically shifted to 1579 cm^−1^ as well as the peak at 1722 cm^−1^, which was apparently weakened, reflecting the interaction between Cd(II) ion and EDTA carboxylate groups. Similar behavior was observed in our previous study for Cd(II) and Pb(II) adsorption onto ethylene glycol-bis(2-aminoethylether)-N,N,N′,N′-tetraacetic acid (EGTA) modified chitosan^25^. Besides, both these varieties were also found for only BPS adsorption, revealing that EDTA groups were also involved in the cationic organic molecule adsorption, consistent with the adsorption isotherm study.

**Figure 9:**
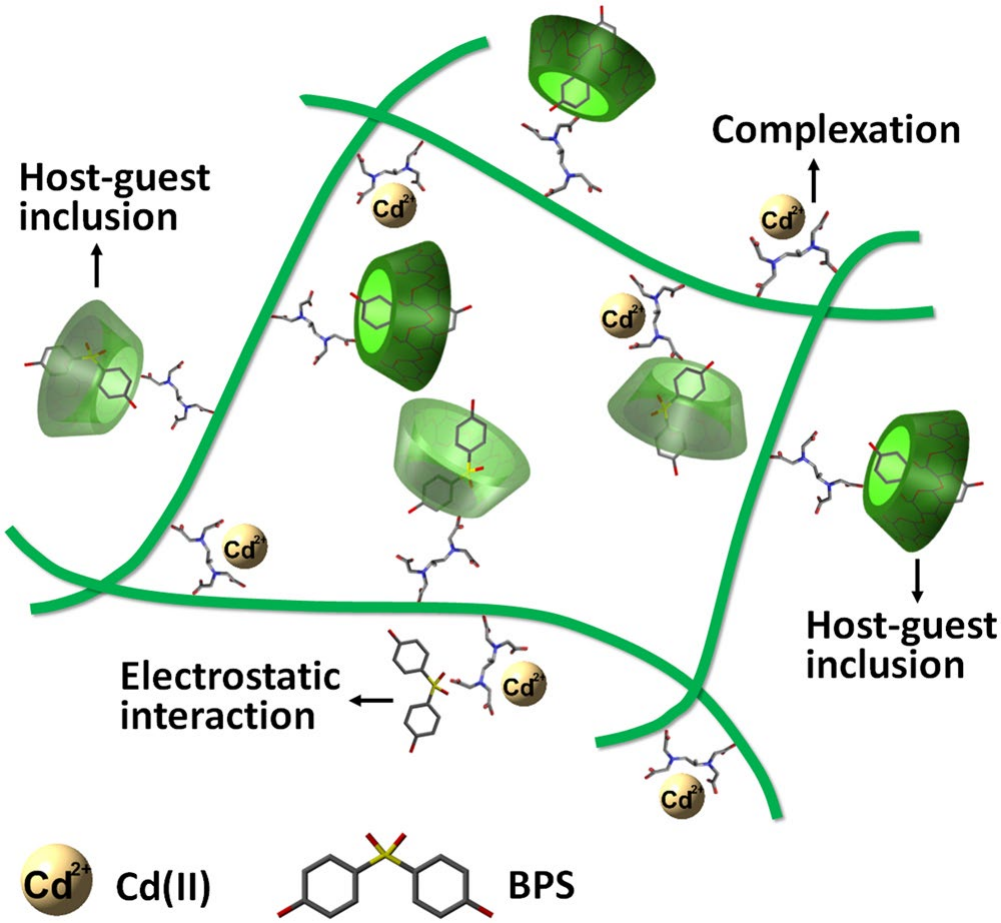
The schematic illustration of the related adsorption mechanisms of CS-ED-CD toward Cd(II) and BPS.

On the basis of isotherms, SEM elemental mapping, and FT-IR studies, a possible adsorption mechanism for the removal of inorganic and organic pollutants is proposed in Fig. 9. The superior sorption onto CS-ED-CD could be ascribed to the following aspects. (1) The EDTA moieties act not only as cross-linkers but also as sorption sites for metal ions complexation. EDTA forms intra- or inter-molecular cross-links with chitosan chains to produce hydrogel. EDTA also interacts with chitosan and amino-CD to immobilize CD cavities on chitosan backbones. The EDTA chelating property endows the polymer with preferential metal sorption ability^9^. (2) The immobilized CD cavities sequester the target organic compound molecules through host-guest inclusion interaction. As shown in Fig. S4, the molecular sizes of the most studied organic pollutants are suitable for β-CD cavity with inner diameter of 0.78 nm^17^. Noticeably, the full size of imipramine (0.94 × 0.97 × 0.58 nm) is somewhat larger than the inner diameter of β-CD cavity. Thus, it is possible that the aromatic rings of imipramine molecules (Fig. S4d) were embedded into CD cavities and the rest branched parts remained outside. This could also explain why imipramine displayed relatively lower sorption capacity than other compounds. (3) The negative-charged COO^−^ groups (from EDTA) within polymer matrix capture the cationic organic pollutant molecules via electrostatic interaction.

### Regeneration

**Figure 10:**
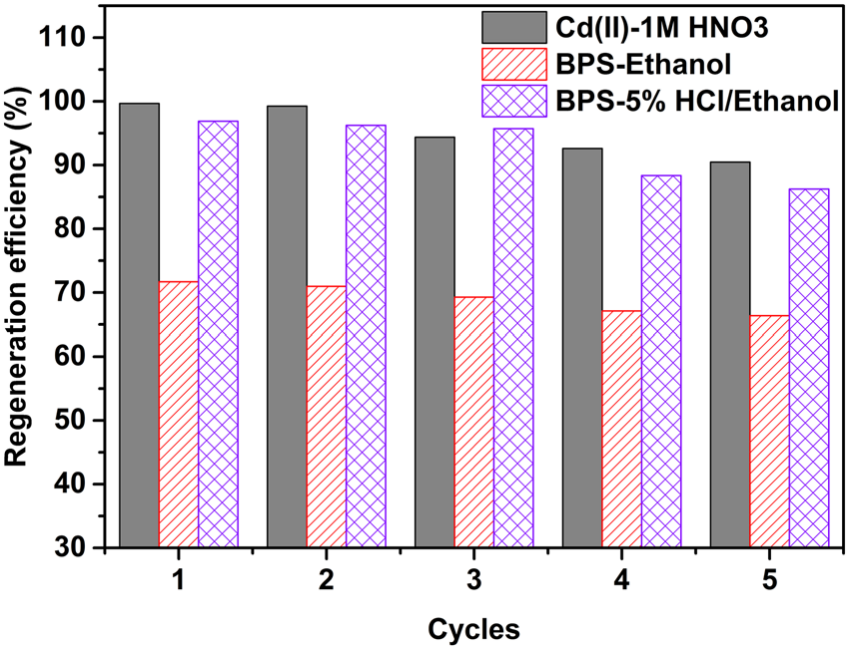
Regeneration of CS-ED-CD for Cd(II) by using 1 M HNO_3_ and for BPS by using ethanol and 5% HCl/ethanol (v/v), respectively (dose, 1 g L^−1^; pH 4; contact time, 360 min; initial concentration, 100 mg L^−1^ Cd(II) and 50 mg L^−1^ BPS).

The reusability is a significant feature of an advanced adsorbent for feasible and practical application. In this study, Cd(II)-loaded CS-ED-CD was regenerated using 1 M HNO_3_ according to the previous regeneration approaches for metal loaded EDTA-modified adsorbents^28,38^. In the case of organic compound-loaded CS-ED-CD, on account of its main sorption mechanism of host-guest inclusion interaction, organic solvents such as ethanol was chosen for organic pollutant desorption^56^. Moreover, EDTA-groups partly participated in the cationic organic compound adsorption, thus 5% HCl in ethanol solution (v/v) was further applied for the regeneration of BPS-loaded CS-ED-CD^28^. Figure 10 shows the regeneration of the adsorbent over five cycles. Evidently, Cd(II)-loaded CS-ED-CD could be successfully regenerated using 1 M HNO_3_, and the regeneration efficiency was 99.67% and 99.25% in the first two cycles and 92.62% and 90.48% in the fourth and fifth cycles, respectively. This also indicated its resistance to extreme pH. It is observed that the BPS loaded adsorbent could not be effectively regenerated by absolute ethanol (≤70%), however, it could be successfully accomplished using 5% HCl in ethanol solution. It was found that the regeneration efficiency was 96.88% and 96.21% in the first two cycles and decreased slightly to 88.36% and 86.24% at the fourth and fifth cycles, respectively. The results revealed the stability and recyclability of CS-ED-CD in practical applications.

### Evaluating the performance of CS-ED-CD in mixture of pollutants at environmentally (μg L^−1^) relevant concentrations

**Figure 11:**
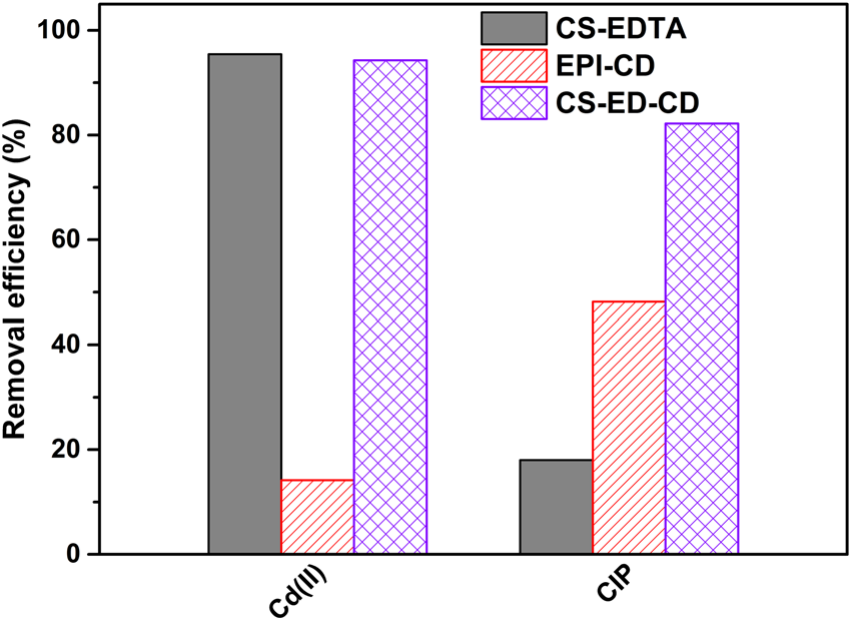
CS-ED-CD outperforms CS-EDTA and EPI-CD for the simultaneous removal of Cd(II) and CIP at environmental concentrations. Initial concentration Cd(II) 100 μg L^−1^ and CIP 50 μg L^−1^; dose 1 g L^−1^; contact time 360 min; limit of detection Cd(II) 1.9 μg L^−1^ and CIP 0.5 μg L^−1^.

The simultaneous removal of inorganic and organic pollutants was also investigated at environmentally relevant (μg L^−1^) concentrations and in a mixture solution of Cd(II) at 100 μg L^−1^ and CIP at 50 μg L^−1^, the concentration which many inorganic and organic micropollutants mostly present in wastewater^57^ and drinking water resources^1^. As shown in Fig. 11, CS-ED-CD performed equal uptake of Cd(II) but with a much larger uptake of CIP than CS-EDTA, whereas both the pollutants showed overwhelming uptake by CS-ED-CD over EPI-CD. These demonstrate that CS-ED-CD could effectively and simultaneously remove inorganic and polar organic pollutants at environmentally relevant concentrations, suggesting that CS-ED-CD serves as a promising adsorbent for practical removal of a wide range of micropollutants from aqueous solutions.

## Conclusions

A novel and environmentally friendly trifunctional adsorbent, CS-ED-CD, was fabricated via a facile one-pot synthesis by using EDTA-groups as cross-linkers. The adsorbent exhibited high absorptivity toward Pb(II), Cd(II), BPS, CIP, procaine, and imipramine with maximum adsorption capacities of 0.803, 1.258, 0.177, 0.142, 0.203, 0.149 mmol g^−1^, respectively. This study, based on the adsorption behavior and characterization results, provides evidence that each component of CS-ED-CD has a crucial role in its functioning: chitosan is the backbone of this novel polymer; importantly, the EDTA-groups play the role not only as cross-linkers but also as complexation sites for metal ions; on the other hand, the adsorption mechanism of organic pollutants onto CS-ED-CD is primarily the host-guest inclusion of CD cavities. Additionally, the adsorbent performed an efficient and simultaneous removal of inorganic and organic pollutants at environmental concentrations. In summary, this work adds a new insight to design and preparation of a trifunctional cyclodextrin-based polymer adsorbent, which can remove targeted inorganic and organic micropollutants from aqueous solution simultaneously. It is believed that this green cross-linking technology can be extended to prepare a wide variety of trifunctional materials for various applications.

## Materials and Methods

### Materials

All reagents were purchased from Sigma-Aldrich (Finland/Canada) and were used without further purification. β-cyclodextrin (β-CD) was 97+% pure, and chitosan flakes 85+% deacetylated had a molecular weight ranging from 190 000 to 375 000 g mol^−1^ and a viscosity of 200–2000 MPa. All other chemicals were analytical grade. All aqueous solutions of pollutants were prepared using 18 MΩ deionized water. The chemical properties of model organic compounds are presented in Table S3. Adjustment of pH was conducted using 0.1 M NaOH/HNO_3_ for metals while 0.1 M NaOH/HCl for organic compounds, respectively.

### Synthesis of amino-β-cyclodextrin (amino-β-CD)

Prior to cross-linking, β-CD was functionalized with amino group via two steps of tosylation and amination, by reference to the previous literatures^23,58^. Briefly, 4.0 g of β-CD and 3.0 g of toluenesulfonyl chloride (TsCl) were dissolved in 50 mL of 0.5 M NaOH solution and reacted under vigorous stirring at 0 °C for 1 h. After the reaction, the unreacted TsCl was filtered off and then the filtrate was neutralized by using 0.1 M HCl, obtaining tosylated β-CD (β-CD-TsCl) as a precipitate. Secondly, the as-prepared β-CD-TsCl (3.0 g) was reacted with 20 mL of ethylenediamine under reflux at 40 °C for 24 h. The amino-modified β-CD (amino-β-CD) was obtained by reprecipitation of the solution from 80 mL of acetone.

### Synthesis of Chitosan-EDTA-β-cyclodextrin (CS-ED-CD)

The as-prepared amino-β-CD (0.5 g) and 1.0 g chitosan were dissolved in 20 mL of 10% (v/v) acetic acid solution and then diluted four times with methanol. Subsequently, as a cross-linking agent, 6.0 g of EDTA dianhydride synthesized according to Repo *et al*.^59^ was suspended in 5 mL methanol, which was added dropwise to the solution. The mixture was stirred at 500 rpm for about 24 h at room temperature. The resulted yellowish gel was filtered and mixed with ethanol under continuous stirring for an additional 5 h. The residual EDTA was removed by washing the gel with an excess of 0.1 M NaOH. Then the product was repeatedly rinsed with deionized water, 0.1 M HCl, and deionized water. Finally, the swollen hydrogel was rapidly frozen in liquid nitrogen and dried in a freeze-dryer (FreeZone, Labconco) under a high vacuum at −42 °C for 72 h and stored in a desiccator until use.

### Synthesis of Chitosan-EDTA (CS-EDTA), EDTA-cross-linked β-cyclodextrin (EDTA-CD), and Epichlorohydrin β-cyclodextrin (EPI-CD)

As a blank control, an insoluble EDTA-cross-linked chitosan (CS-EDTA) and an EDTA-cross-linked β-cyclodextrin (EDTA-CD) were synthesized according to our previous reports^28,38^. Meanwhile, for comparison purpose, an epichlorohydrin cross-linked β-CD (EPI-CD), which is the most widely studied β-CD polymer that has been commercialized for water treatment^60^, was also synthesized according to the typical procedures^16^.

### Material characterization

The Fourier transform infrared (FTIR) spectroscopy of the type VERTEX 70 (Bruker, Germany) with a platinum ATR attachment was employed to identify the functional groups of the materials. To investigate the elemental distribution on the surface of CS-ED-CD after adsorption, Quantitative analyses of the contents of each component in CS-ED-CD polymer were performed with a 2400 Series II CHNS/O Analyzer (PerkinElmer, U.S.A.). The amount of carboxylic acid groups on CS-ED-CD was evaluated via Conductometric-potentiometric titration method using a Metrohm 809 Titrando autotitrator (Switzerland). The amount of active β-CD cavities on CS-ED-CD was determined through the Photometric Titration with UV-vis spectrometry (PerkinElmer Lambda 45, U.S.A.) using phenolphthalein as indicator. The surface and cross-sectional morphologies of the as-prepared polymer were investigated using a Jeol JSM-5800 (Japan) scanning electron microscope (SEM) at an acceleration voltage of 5.0 kV. The surface area, pore size and cumulative pore volume of the synthesized CS-ED-CD were examined at 77.35 K using Brunauer-Emmett-Teller (BET) surface area analyzer (Tristar®II Plus) with nitrogen adsorption isotherms measured in the range of relative pressures from 0.0 to 1.0. Elemental Mapping was performed during the SEM examination (acceleration voltage 20.0 kV) by Thermo Scientific Ultra Dry SDD Energy-dispersive X-ray spectroscopy (EDS). The ζ-potential of the adsorbent was measured using a Zetasizer Nano ZEN3500 (Malver, U.K.). Thermogravimetric analysis (TGA) and derivative thermogravimetric (DTG) tests were conducted using a NETZSCH TG 209F1 (Germany) at a heating rate of 10 °C min^−1^ under a nitrogen atmosphere from 25 °C to 1000 °C.

### Adsorption Experiments

All adsorption experiments were conducted by mixing 10 mg of adsorbents with 10 mL of pollutant solutions. The effect of pH was investigated at an initial concentration of 50 mg L^−1^ in the pH range of 1–6 for metals and pH of 2–10 for organic pollutants, respectively. Adsorption kinetics were performed at time intervals ranging between 5 and 300 min at metal concentrations of 50 mg L^−1^ and organic pollutant concentrations of 25 mg L^−1^, respectively. After each experiment, the adsorbents were separated from solutions using a 0.45 μm polypropylene syringe filter. The concentrations of initial organic pollutants as well as in the filtrates were determined by UV-vis spectrometry (PerkinElmer Lambda 45, U.S.A.) at their maximum absorbance (Table S3). After dilution with 5% HNO_3_, the metal concentrations before and after adsorption were analyzed by an inductively coupled plasma optical atomic emission spectrometry (ICP-OES) Model Icap 6300 instrument (Thermo Electron Corporation, U.S.A.).

The removal efficiency of pollutant (in %) by the adsorbent was calculated using the following equation:

4 $${\boldsymbol{R}}{\boldsymbol{ \% }}{\boldsymbol{=}}\frac{{{\boldsymbol{C}}}_{{\bf{0}}}-{{\boldsymbol{C}}}_{{\boldsymbol{t}}}}{{{\boldsymbol{C}}}_{{\bf{0}}}}\times {\bf{100}}{\boldsymbol{ \% }}$$

where *R*% is the removal efficiency, whereas *C*_0_ and *C*_*t*_ are the initial and residual concentrations (mmol L^−1^) of pollutants, respectively.

The adsorption capacities (mmol g^−1^) of adsorbents were calculated from the following equation:

5 $${{\boldsymbol{q}}}_{{\boldsymbol{e}}}=\frac{({{\boldsymbol{C}}}_{0}-{{\boldsymbol{C}}}_{{\boldsymbol{t}}})}{{\boldsymbol{M}}}{\boldsymbol{V}}$$

where *C*_0_ and *C*_*t*_ are the initial and residual concentrations (mmol L^−1^) of pollutants, respectively, whereas *M* (g) and *V* (L) are the weight of the adsorbents and the volume of the solutions, respectively.

### Regeneration experiments

To investigate the reusability of CS-ED-CD, the desorption of Cd(II) and BPS was performed as models for inorganic pollutants and organic pollutants, respectively. Firstly, 50 mg of dry CS-ED-CD adsorbent was mixed with 50 mL of 100 mg L^−1^ Cd(II) or 50 mL of 50 mg L^−1^ BPS solution for 360 min to reach saturation. Then the adsorbents were separated and regenerated by soaking them in 10 mL of 1 M HNO_3_ for Cd(II), and in either 10 ml of absolute ethanol or 5% HCl in ethanol (v/v) for BPS. After 10 min soaking, the adsorbents were filtered and washed with deionized water and reconditioned for sorption in subsequent cycles.

### Evaluating the performance of CS-ED-CD in mixture of pollutants at environmentally (μg L^−1^) relevant concentrations

100 mg of the adsorbent (CS-EDTA, EPI-CD, or CS-ED-CD) was mixed with 100 mL of the diluted mixture (100 μg L^−1^ Cd(II) and 50 μg L^−1^ CIP). After a 360 min contact at room temperature, the adsorbent was separated using a 0.45 μm polypropylene syringe filter and the residual Cd(II) concentrations were analyzed by ICP-OES, as well as the CIP concentrations in filtrates were analyzed by ultra-high performance liquid chromatography (UHPLC) coupled with a Xevo TQ mass spectrometer (MS) (Waters, UK). Deuterated ciprofloxacin (^2^H_8_) was used as an internal standard. Samples were injected and loaded onto an Acquity UPLC BEH (Waters) C18 Column (2.1 mm × 50 mm, particle size 1.7 µm). Elution was performed with mobile phase A, containing of 0.1 vol.% formic acid in water, and mobile phase B of methanol. Flow rate was 0.5 mL min^−1^. The MS was operated in multiple reaction monitoring (MRM) mode of channel ES+. The quantifier MRMs were 332.2 > 231.1 for ciprofloxacin and 340.2 > 235 for deuterated ciprofloxacin. Retention times were 3.48 for ciprofloxacin and 3.47 min for deuterated ciprofloxacin, respectively. The limit of detection for ciprofloxacin was determined as the lowest calibration concentration which has the relative standard deviation below 10%. The limit of detection for Cd(II) was calculated as three times the standard deviation of ten runs of the blank solutions according to literature^29^.

## Electronic supplementary material

Supplementary Information
