# Supplementary material for: One-pot synthesis of trifunctional chitosan-EDTA-β-cyclodextrin polymer for simultaneous removal of metals and organic micropollutants
Source: Sci Rep. 2017 Nov 17;7:15811. doi: 10.1038/s41598-017-16222-7 (PMC5693995; doi:10.1038/s41598-017-16222-7)
Supplement: Supplementary file 1 — Supplementary Information [file 41598_2017_16222_MOESM1_ESM.doc]

Supplementary information

**One-pot synthesis of trifunctional chitosan-EDTA-β-cyclodextrin polymer for simultaneous removal of metals and organic micropollutants**

Feiping Zhao, Eveliina Repo, Dulin Yin, Li Chen, Simo Kalliola, Juntao Tang, Evgenia Iakovleva, Kam ChiuTam, Mika Sillanpää

**The following supplementary information includes 9 supporting pages, 4 supporting figures and 3 supporting tables:**

- Fig. S1. Effect of pH on adsorption of Cd(II) (a), CIP (b), Procaine (c), and Imipramine (d) by CS-ED-CD. Experimental conditions: dose 1 g L^-1^, contact time 360 min, initial concentration of target pollutant: 50 mg L^-1^.
- Fig. S2. UV-vis spectra of organic pollutants at varying contact time and pseudo-second-order plots for organic pollutants and metals by CS-ED-CD.
- Fig. S3. FTIR spectra of BPS and CS-ED-CD before and after Cd(II) and/or BPS adsorption.
- Fig. S4. The 3D-structures and dimensions of BPS (a), CIP (b), procaine (c), imipramine, and its branched part (d).
- Table S1. Experimental conditions and yields for the synthesis of EPI-CD, CS-EDTA, EDTA-CD, and CS-ED-CD.
- Table S2. Elemental analysis results of CS-ED-CD polymer and monomers.
- Table S3. Name, CAS number, chemical structure of the target organic compounds.


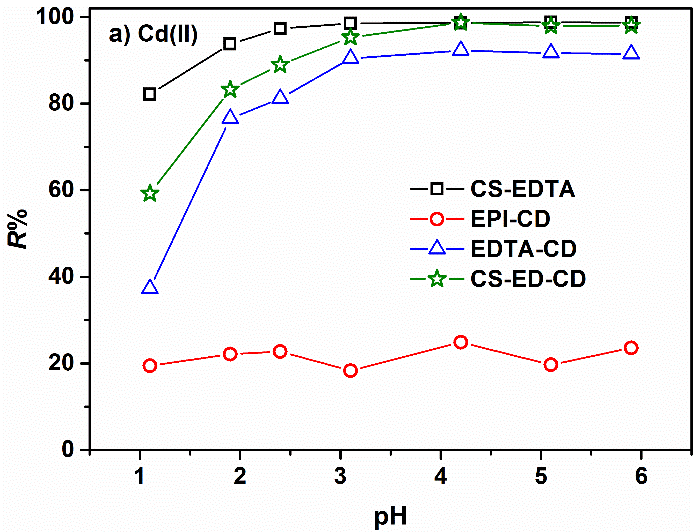

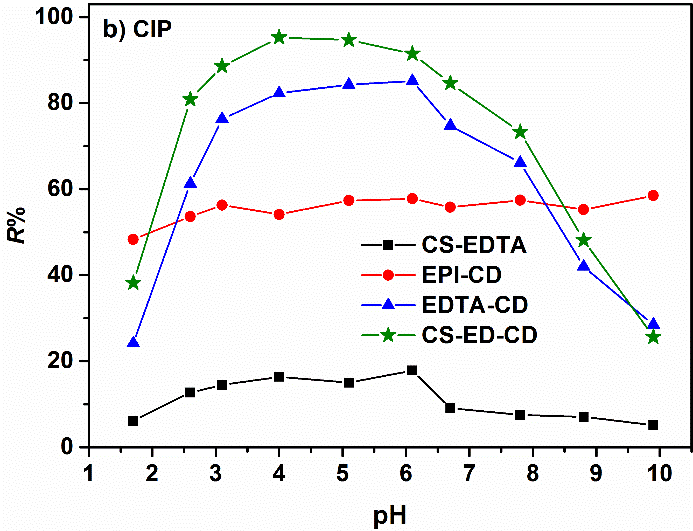

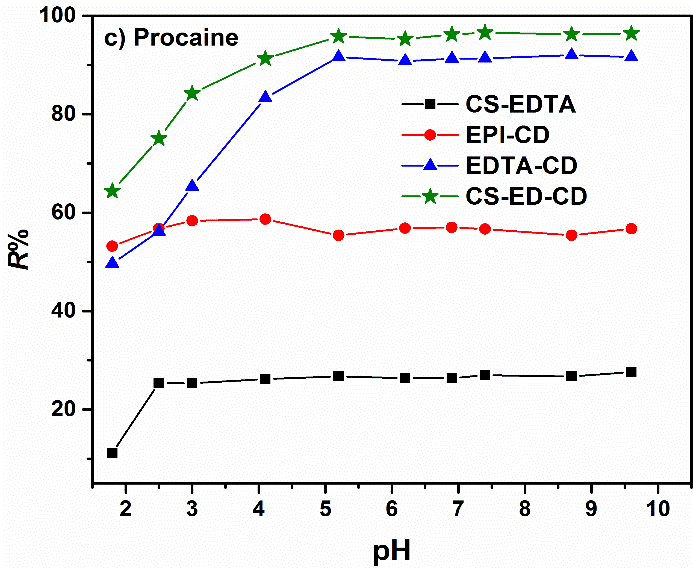

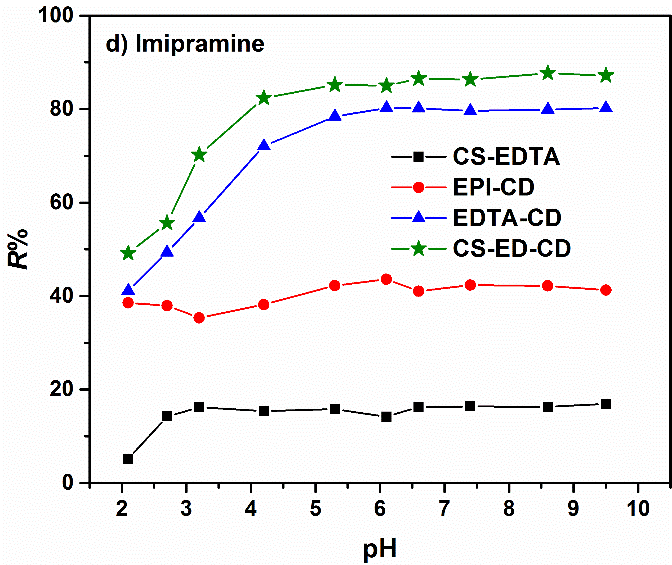


**Figure S1.** **Effect of pH on adsorption of Cd(II) (a), CIP (b), Procaine (c), and Imipramine (d) by CS-ED-CD. Experimental conditions: dose 1 g L^-1^, contact time 360 min, initial concentration of target pollutant: 50 mg L^-1^.**


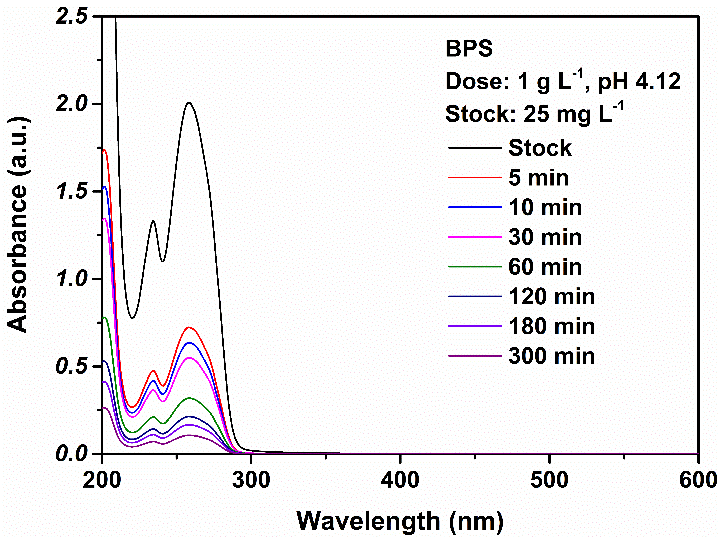

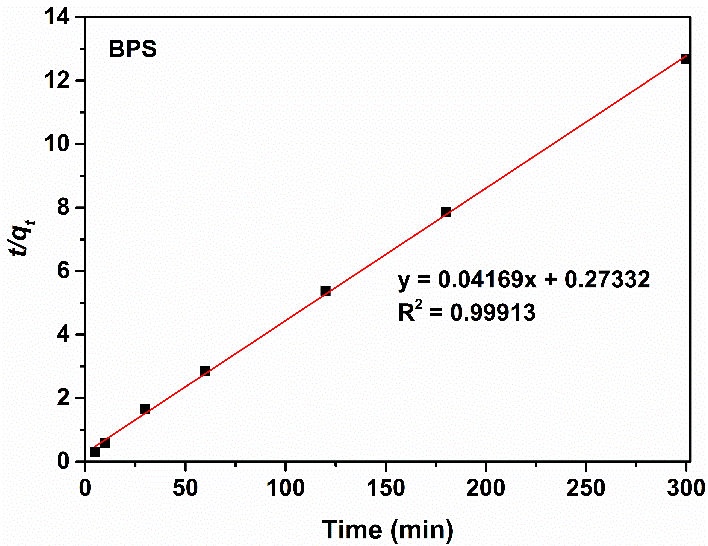


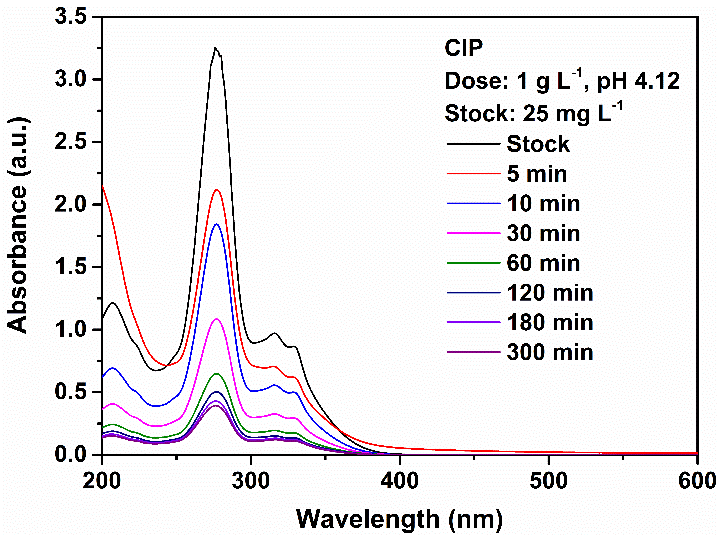

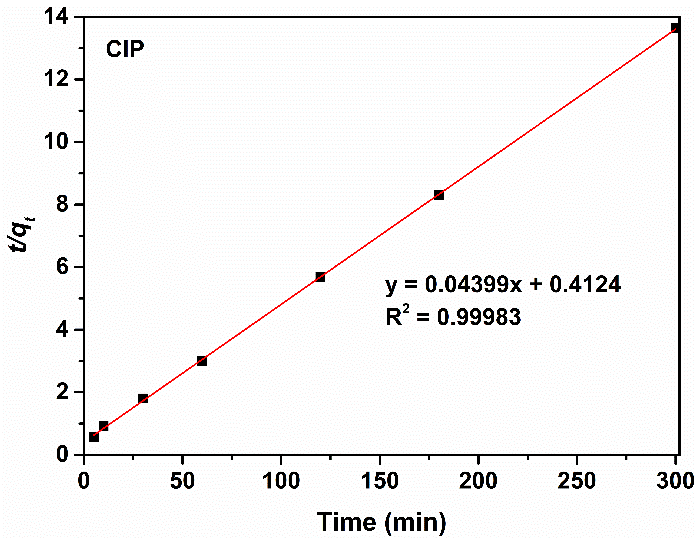

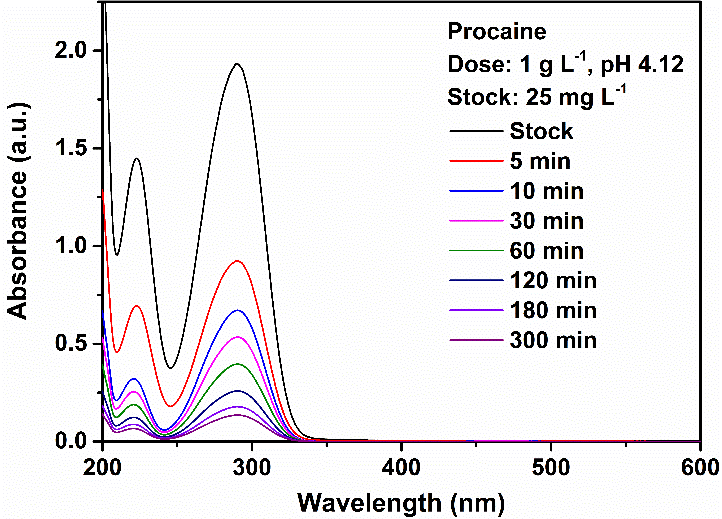

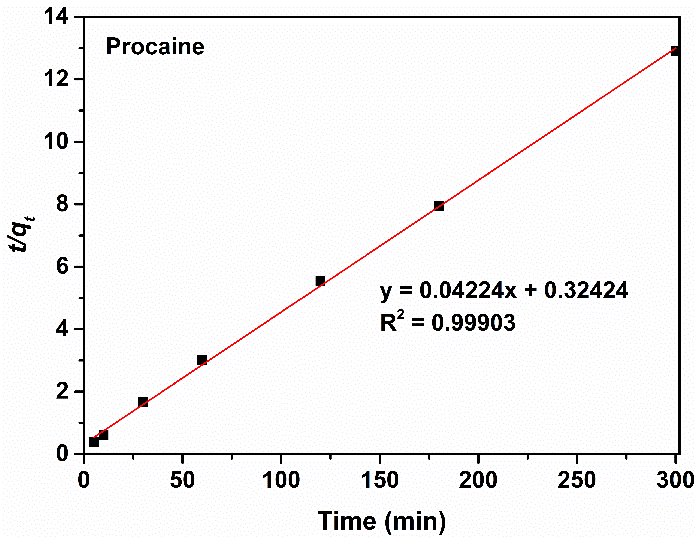


**Figure S2. UV-vis spectra of organic pollutants at varying contact time (colored traces) and pseudo-second-order plots (linear) for organic pollutants and metals by CS-ED-CD.**

(Continue on next page)

(Continued from last page)


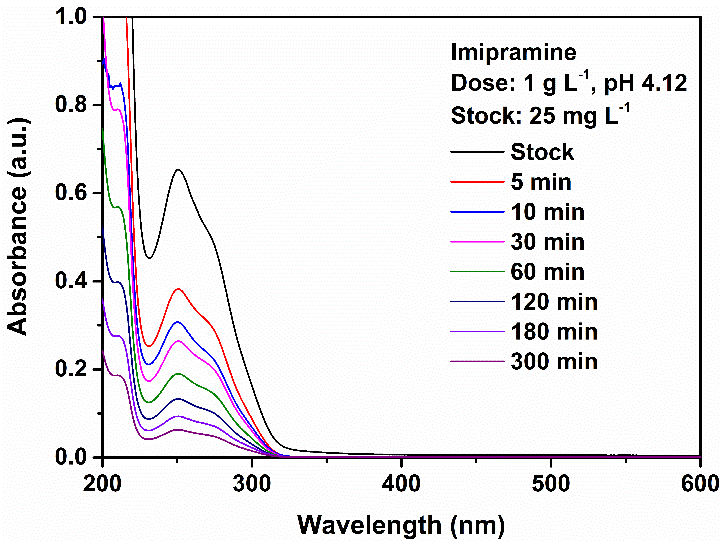

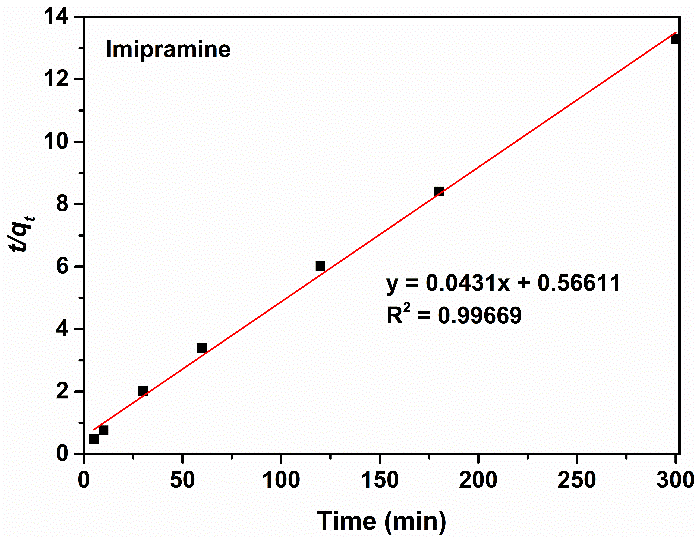


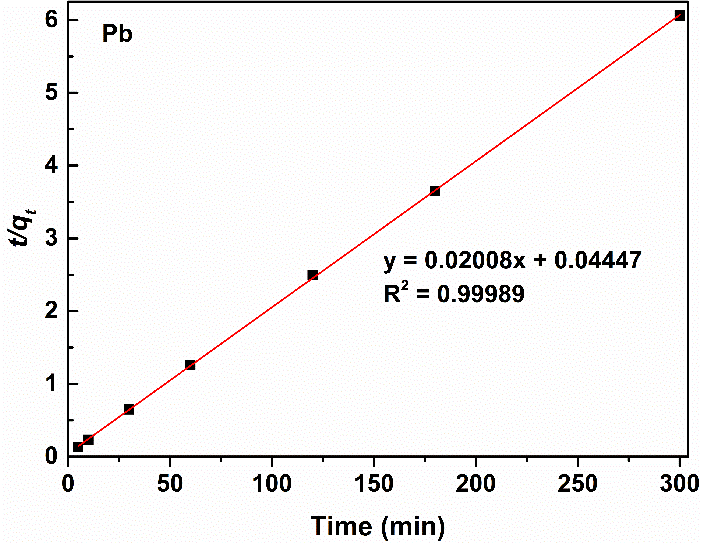

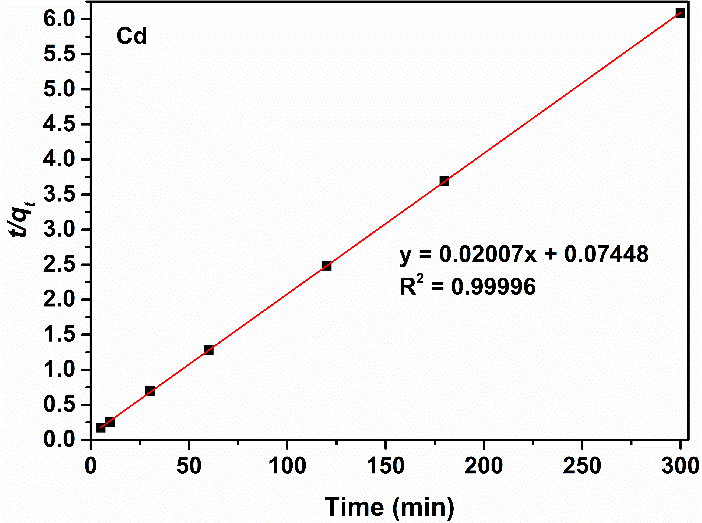


**Figure S2. UV-vis spectra of organic pollutants at varying contact time** (colored traces) **and pseudo-second-order plots** (linear) **for organic pollutants and metals by CS-ED-CD.**


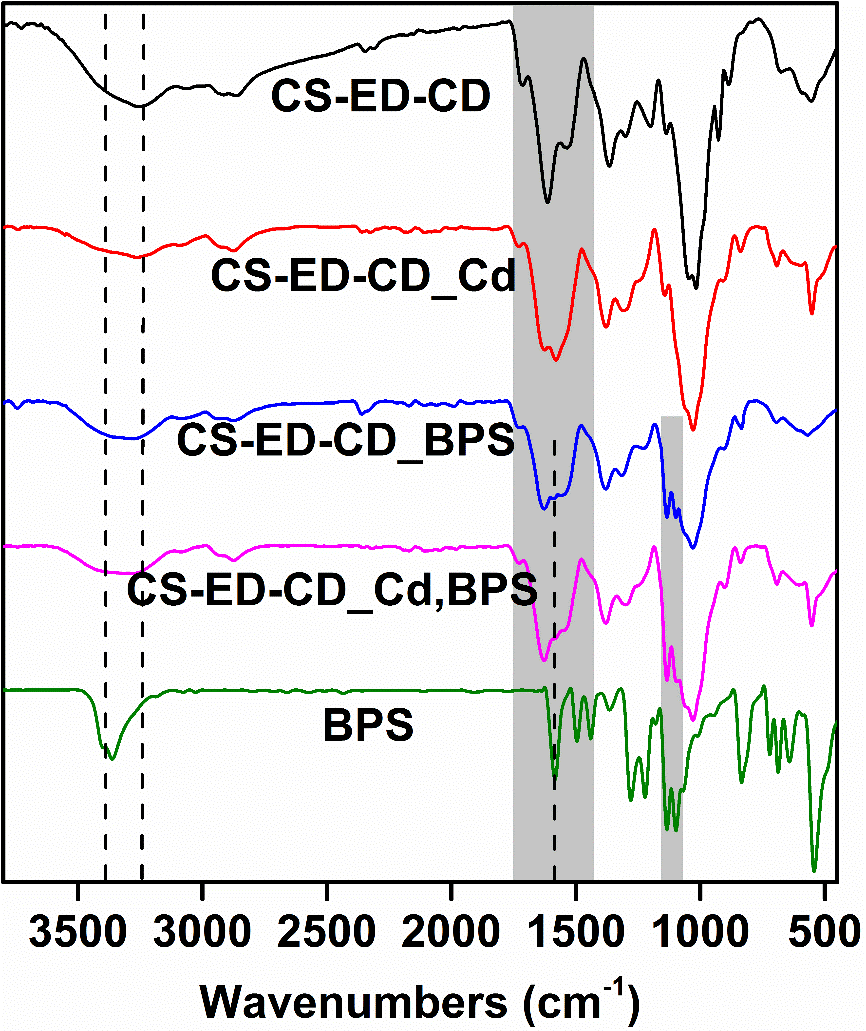


**Figure S3.** **FTIR spectra of BPS and CS-ED-CD before and after Cd(II) and/or BPS adsorption.** (initial concentration: 100 mg L^-1^).


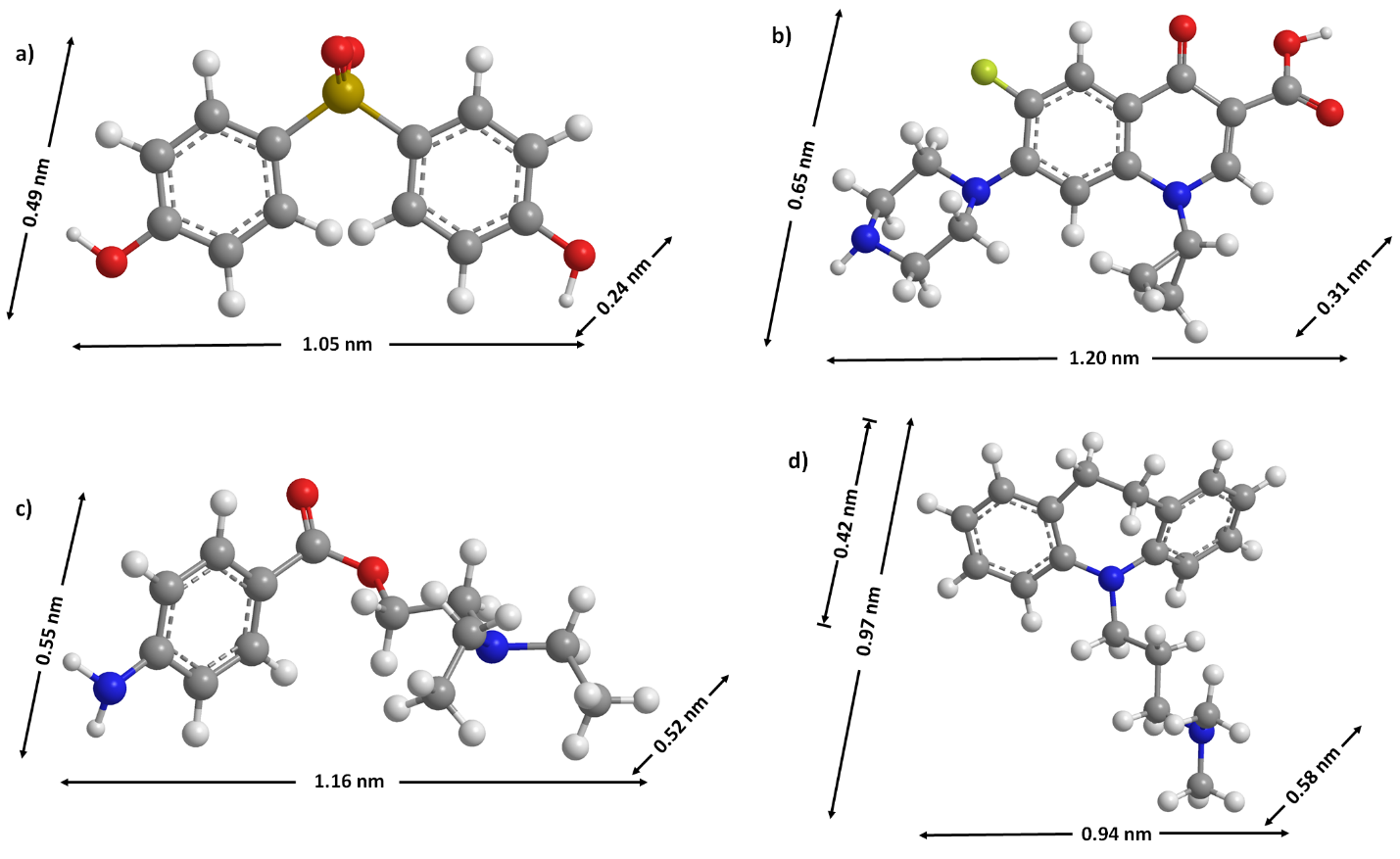


**Figure S4.** **The 3D-structures and dimensions of BPS (a), CIP (b), procaine (c), imipramine, and its branched part (d).** The structures were optimized with Chem-Bio 3D software employing MMFF94 energy minimization method.

**Table S1. Experimental conditions and yields for** the synthesis of EPI-CD, CS-EDTA, EDTA-CD, and CS-ED-CD.

| Samples | Chitosan (g) | β-CD/Amino-β-CD (g) | EDTA/EDTAD/EPI (g) | Product (g) | Yield (%) |
| --- | --- | --- | --- | --- | --- |
| EPI-CD | - | 4.0 | 3.0 | 4.73 | 67.57 |
| CS-EDTA | 1.0 | - | 6.0 | 1.66 | 23.71 |
| EDTA-CD | - | 4.0 | 6.0 | 0.72 | 7.20 |
| CS-ED-CD | 1.0 | 0.5 | 6.0 | 2.15 | 31.33 |

**Table S2.** **Elemental analysis results of CS-ED-CD polymer and monomers.**

| **Sample** | **Elemental content (wt.%)** | | | | **Proportion in CS-ED-CD** | **Content in CS-ED-CD** | |
| --- | --- | --- | --- | --- | --- | --- | --- |
|  | **N** | **C** | **H** | **S** |  | **wt.%** | **mmol g^-1^** |
| Chitosan | 6.68 | 41.10 | 6.96 | 0 | 0.2153*^X^* | 20.75 | - |
| EDTA | 8.27 | 39.73 | 5.37 | 0 | 0.6198*^Y^* | 59.73 | 2.046^a^ |
| β-CD | 0 | 38.80 | 6.32 | 0 | - |  | - |
| Amino-β-CD | 3.98^b^ | 42.54 | 6.73 | 0 | 0.2026*^Z^* | 19.52 | 0.162^c^ |
| CS-ED-CD | 7.37 | 42.09 | 6.19 | 0 | 1.0377 | 100.00 | - |

*^X^*,*^Y^*,*^Z^* Calculated according to the difference of N, C, and H contents between CS-ED-CD polymer and its monomers as follows:

Set the proportions of chitosan, EDTA, and amino-β-CD in CS-ED-CD polymer are *X*, *Y*, and *Z*, respectively. The mass balance for each of the element is described by ternary linear equations below for N, C, and H:

Nitrogen: 6.68 × *X* + 8.27 × *Y* + 3.98 × *Z* = 7.37 (S1)

Carbon: 41.10 × *X* + 39.73 × *Y* + 42.54 × *Z* = 42.09 (S2)

Hydrogen: 6.96 × *X* + 5.37 × *Y* + 6.73 × *Z* = 6.19 (S3)

So, *X* = 0.2153; *Y* = 0.6198; *Z* = 0.2026

(*X* + *Y* + *Z* = 1.0377)

^a^ molar amount of EDTA group (C_10_H_16_N_2_O_8_, 292 g mol^-1^): (59.73% × 1000 mg g^-1^) ÷ (292 g mol^-1^);

^b^ the as-prepared amino-β-CD was defined based on N% (3.98%): the stoichiometric N% value of β-CD-(NHCH_2_CH_2_NH_2_) (C_44_H_77_N_2_O_34_) is 2.38%, while the stoichiometric N% value of β-CD-(NHCH_2_CH_2_NH_2_)_2_ (C_44_H_77_N_2_O_34_) is 4.59%, so the as-prepared amino-β-CD can be accurately defined as β-CD-(NHCH_2_CH_2_NH_2_)_1.72_ (1208.96 g mol^-1^).

^c^ molar amount of amino-β-CD group (β-CD-(NHCH_2_CH_2_NH_2_)_1.72_, 1208.96 g mol^-1^): (19.52% × 1000 mg g^-1^) ÷ (1208.96 g mol^-1^).

**Table S3. Name, CAS number, chemical structure of the target organic compounds.**

| **Substance** | **CAS No.** | **Chemical structure** | | **Ionization mode** | **Use** | **λ_max_ (nm)** |
| --- | --- | --- | --- | --- | --- | --- |
| Bisphenol-S (BPS) | 80-09-1 | C_12_H_10_O_4_S,  MW 250.3 | 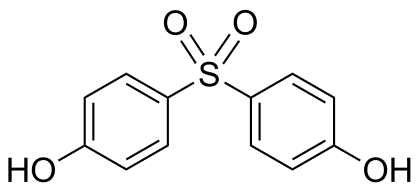 | Positive | Endocrine disruptor | 258 |
| Ciprofloxacin (CIP) | 85721-33-1 | C_17_H_18_FN_3_O_3_,  MW 331.3 | 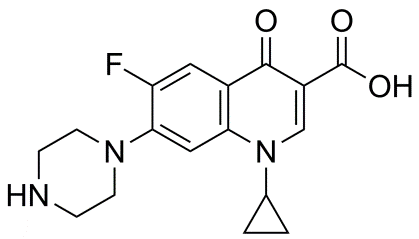 | Zwitterionic | Antibiotic | 277 |
| Procaine | 59-46-1 | C_13_H_20_N_2_,  MW 236.3 | 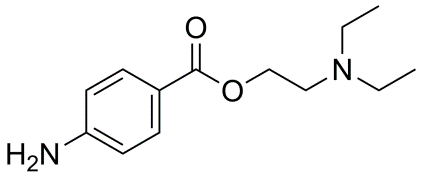 | Positive | Anesthetic | 290 |
| Imipramine | 50-49-7 | C_19_H_24_N_2_,  MW 280.4 | 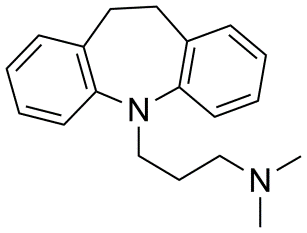 | Positive | Antidepressant | 249 |
